# Supplementary material for: Modulation of signaling cross-talk between pJNK and pAKT generates optimal apoptotic response
Source: PLoS Comput Biol. 2022 Oct 14;18(10):e1010626. doi: 10.1371/journal.pcbi.1010626 (PMC9604984; doi:10.1371/journal.pcbi.1010626)
Supplement: S1 Text — (PDF) [file pcbi.1010626.s001.pdf]

Modulation of signaling cross-talk between pJNK and pAKT generates optimal apoptotic response

Sharmila Biswas[1,¶] , Baishakhi Tikader[2,¶], Sandip Kar[2*], Ganesh A Viswanathan[1*]

[1]Department of Chemical Engineering, Indian Institute of Technology Bombay, Mumbai, India.

[2]Department of Chemistry, Indian Institute of Technology Bombay, Mumbai, India.

[¶]These authors contributed equally to this work

[*]Corresponding authors

E-mail: sandipkar@iitb.ac.in, ganeshav@iitb.ac.in

S1 Text

Apoptosis and intracellular marker protein level detection

S1.1: List of reagents

Sr No.	Reagents	Company
1.	RPMI-1640	HiMedia (Mumbai, India)
2.	fetal bovine serum (FBS)	HiMedia (Mumbai, India)
3.	L-glutamine	HiMedia (Mumbai, India)
4.	Antibiotic–antimycotic solution	HiMedia (Mumbai, India)
5.	TNFα	Peprotech
6.	Triptolide (TPL)	Sigma-Aldrich
7.	Dimethyl sulphoxide (DMSO)	HiMedia (Mumbai, India)
8.	Paraformaldehyde	HiMedia (Mumbai, India)
9.	Dulbecco's Phosphate Buffered Saline 1X	HiMedia (Mumbai, India)
10.	Alexa dyes	ThermoFischer Scientific
11.	Bovine Serum Albumin (BSA)	HiMedia (Mumbai, India)
12.	phospho-pAKT(pS473)- Alexa 488-tagged	BD Biosciences
13.	phospho-pJNK(pT183/pY185)- PE tagged	BD Biosciences
14.	anti-active Caspase3- V450 tagged	BD Biosciences
15.	FITC-labeled Annexin V and PI	BD Pharmingen, San Diego, CA, US
16.	Wortmannin	Sigma-Aldrich
17.	SP600125	Sigma-Aldrich

S1.2: Apoptosis detection

Apoptotic cells display phosphatidylserine (PS) on their membrane surface. Annexin-V conjugated with FITC has high binding affinity towards PS, thus cells which are programmed to undergo cell death will show FITC positive. However, cells with intact cellular membrane will repel the PI stain. Thus, the live quadrant shows both FITC and PE negative as cells are live and will not display PS on its cell surface membrane. Early apoptotic cells will display PS on its surface but have intact cellular membrane thus will show FITC positive and PE negative. Late apoptotic cells will display both PS on its surface as well will have disrupted cellular membrane showing both FITC and PE

positive. Finally, necrotic cells will show no display of PS on its surface but have disrupted cellular membrane hence will only show PE positive. In Fig I, we show a sample four-quadrant Annexin-V vs PI plot corresponding to the population of U937 cells, stimulated with TNFα for 24 h, distributed among these quadrants. We considered the sum of both Early and Late apoptotic cells (quadrants 2 and 3) as the total percentage of cells undergoing apoptosis. Compensation controls included were untreated-unstained-cells (Fig II-i), untreated cells stained with both Annexin-V and PE (Fig II-ii), treated cells stained only with Annexin-V and treated cells stained only with PE.

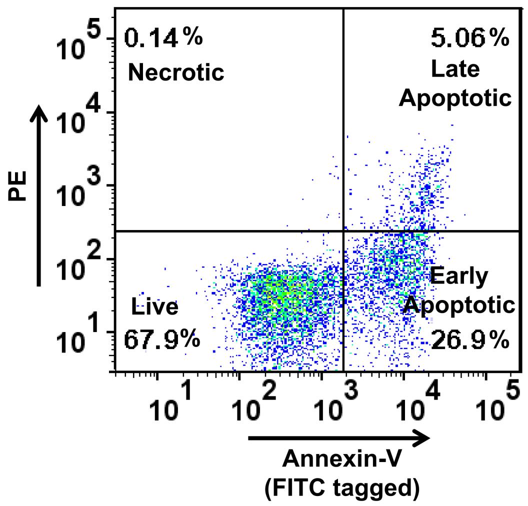

Fig I. Fraction of U937 cell population stimulated with TNFα for 24 h undergoing apoptosis. The four-quadrant plot depicting cells at different stages of cell death. Annexin-V is tagged with FITC and PI dye is detected in PE channel.

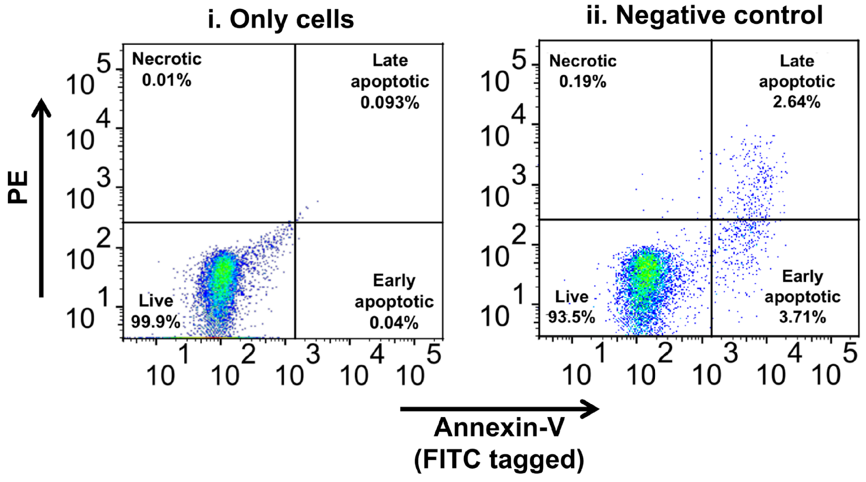

Fig II. Fraction of untreated U937 cell population i. without dye (only cells) and ii. with both the dyes (Negative control). The four-quadrant plot depicting cells at different stages of cell death. Annexin-V is tagged with FITC and PI dye is detected in PE channel.

S1.3: Intracellular protein marker detection

The three marker proteins pAKT, pJNK, and Caspase3 are detected by tagging them with respective fluorescent dye tagged monoclonal antibody. The respective emitted fluorescence at single-cell level was detected in the appropriate channel in a flow cytometer (BD FACS Aria). Figure III shows the histograms of the fluorescence emitted by these three markers at various time points for U937 cells stimulated with TNFα. The median fluorescence intensity (MFI) were estimated from these histograms. The relative fold change (FC) (Eq. 4, Methods M4, main text) is given by

$$FC = \frac{MFI_t - MFI_{DN}}{MFI_{Neg} - MFI_{DN}} \quad [S1.1]$$

and is used for further analysis. Note that antibodies tagged to different fluorochrome were selected based on the emission and excitation spectra such that it showed minimum spectral overlap among the samples. However, to correct the spectral overlap, if any, five compensation controls were used. These are untreated-unstained-cells, untreated cells stained with all monoclonal antibodies tagged with respective fluorochrome and one sample each containing treated cells with individual monoclonal antibody tagged with the corresponding fluorochrome.

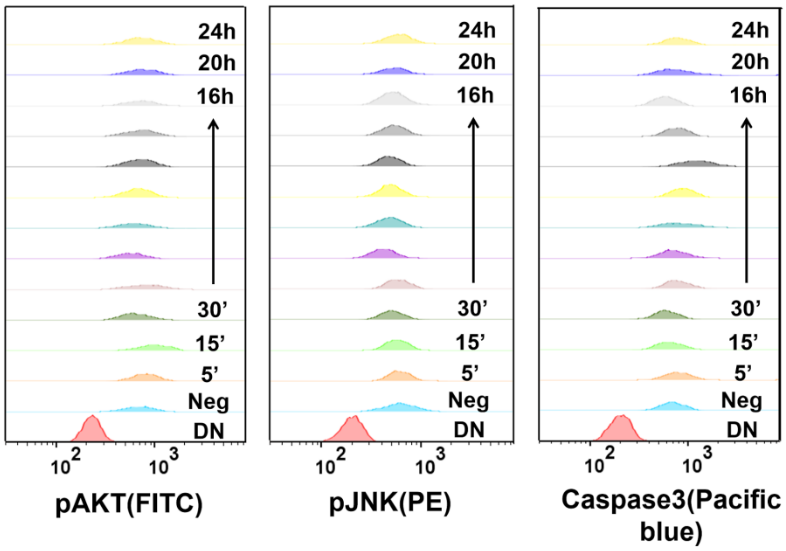

Fig III. Time evolution of the histogram of fluorescence of pAKT, pJNK, and Caspase3 in U937 cells stimulated with TNFα. The emission from the fluorescence tagged monoclonal antibody against pAKT, pJNK and caspase3, respectively were detected in FITC, PE and Pacific blue channels.

S1.4: Untreated experimental controls over 24h time period

Untreated cells were measured over a 24h time period to detect the basal level of the three marker proteins pAKT, pJNK, and Caspase3. Fig IV shows the dynamic fold change (FC) of the signaling proteins under no stimulation condition.

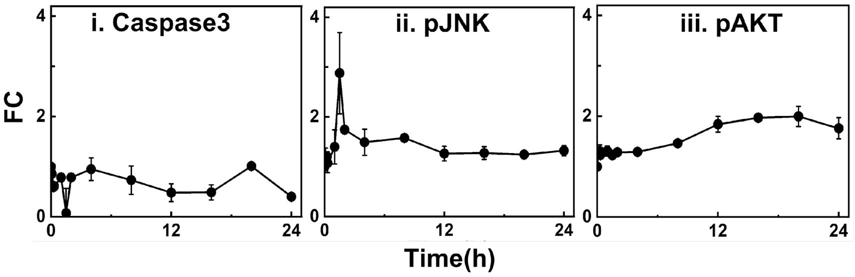

Fig IV. Intracellular signaling patterns of untreated controls: Dynamic trajectories of relative fold change (FC) of (i) Caspase3, (ii) pJNK, and (iii) pAKT at basal levels.

S1.5: Effect of TPL on cell survival

In order to assess the effectiveness of TPL treatment, we estimated the extent of cell survival on different doses of TPL (Fig VA). The effective dose is 60 nM to achieve significant blocking of the survival signaling. We next assessed the sensitivity to the duration of TPL exposure to sufficiently arrest survival signaling. Figure VB shows that 1 h TPL pre-treatment suffices to significantly reduce the survival signaling, which indirectly suggests transactivation of NFκB has been blocked effectively over the experimental duration.

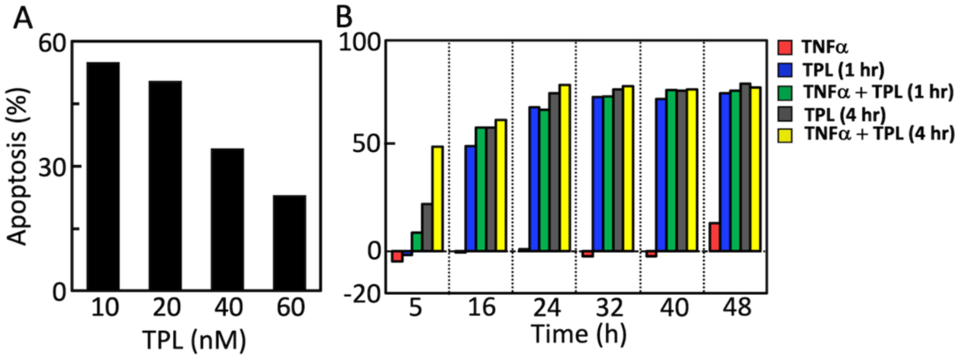

Fig V: Effect of TPL on cell survival. (A) Dose-response of TPL on cell-survival. (B) Effect of TPL pre-treatment duration on apoptosis. Note that negative Apoptosis % in (B) for a few cases are due to negative control (only cells) being more than that when treated.
