# Supplementary material for: Modulation of signaling cross-talk between pJNK and pAKT generates optimal apoptotic response
Source: PLoS Comput Biol. 2022 Oct 14;18(10):e1010626. doi: 10.1371/journal.pcbi.1010626 (PMC9604984; doi:10.1371/journal.pcbi.1010626)
Supplement: S2 Text — (PDF) [file pcbi.1010626.s002.pdf]

Modulation of signaling cross-talk between pJNK and pAKT generates optimal apoptotic response

Sharmila Biswas[1,¶] , Baishakhi Tikader[2, ¶], Sandip Kar[2*], Ganesh A Viswanathan[1*]

[1]Department of Chemical Engineering, Indian Institute of Technology Bombay, Mumbai, India.

[2]Department of Chemistry, Indian Institute of Technology Bombay, Mumbai, India.

[¶]These authors contributed equally to this work

[*]Corresponding authors

E-mail: sandipkar@iitb.ac.in, ganeshav@iitb.ac.in

S2 Text

Detailed model of the TNFα signaling network capturing the cross-talk between different entities

S2.1: Detailed signaling network

TNFα signaling network shown in Fig 2A, main text consists of several interactions whose detailed underlying biochemical reaction along with the corresponding kinetic parameters are depicted in S2 Fig below. We next discuss the network curation along with the biochemical details.

TNFα binds to its receptor TNFR1 transitioning it into its activated form $TNFR1_a$, which subsequently activates several downstream pathways. We included TNFα induced activation of RAF, MKK4/7, NFκB, Ceramide (Cer), PI3K, and Caspase3. For the sake of brevity, in this section, MKK will be used to refer to MKK4/7. The active form of MKKa further activate JNK [1]. Further, RAFa kinase causes the transition between ERK and pERK [1]. Apart from MKK assisted pJNK activation, active ceramide is involved in C1P mediated activation of pJNK [2,3]. PI3K activates ERK [4]. pERK negatively influences its own activation by inhibiting RAF activity [1]. JNK, ROS and MKK are locked in a positive feedback loop [5]. While pJNK is inhibited by pERK [6], in turn, pERK is activated by pJNK. pERK is involved in inhibition of Caspase3 activation. This inhibition occurs via phosphorylation of Caspase9 at Thr 125 causing its inactivation [7]. pJNK regulates Caspase3 activation in a context-dependent manner [8,9].

TNFα mediated cell-survival regulation was incorporated by anti-apoptotic and survival signaling pathways downstream of NFκB [10]. Inhibition of NFκB transactivation using Triptolide (TPL) is included in the network [11]. The anti-apoptotic arm of NFκB signaling was phenomenologically introduced by its negative regulation of Caspase3 activity [12]. Apart from caspases, NFκB also influences pJNK and pAKT. NFκB activates XIAP-and-Gadd45B (XG), which in turn inhibits pJNK [13]. However, NFκB negatively and positively influences pAKT through different pathways. This activation is due to NFκB downregulating PTEN leading to increased pAKT levels via PI3K [14,15,16]. On the other hand, NFκB transcriptionally inhibits Bcl2, which results in further inhibition of pAKT [17,18]. As PTEN is a well-known tumor suppressor gene, its action of inhibiting NFκB activity was included [19]. Active Ceramide negatively regulates pAKT via CAPP [20,21]. By phosphorylating pro-Caspase9 at Ser 196 leading to its inactivation, pAKT causes Caspase3 inhibition [22]. Moreover, pAKT downregulates the activated form of RAF and MKK [23–25].

S2.2: Quantitative dynamic modeling of the TNFα signaling network

We model each of the interactions (Table I) in the biochemical network (S2 Fig) with either law of mass action kinetics or phenomenological functions. Literature support for the mode of action chosen for the interactions are provided in Table I. Using mass balance, we constructed the 16 variable ordinary differential equation based mathematical model of the TNFα network (S2 Fig) constrained by a few algebraic conservation relations shown in S1 Table. Those terms involving phenomenological relationship are discussed in section S2.3 below. Entities involved in the network are defined in S2 Table. The parameters involved in the model are defined in S3 Table.

Table I. Interactions in the TNFα signaling network along with the corresponding biochemical mode of action. Arrows and hammers, respectively represent activation and inhibition.

Sr. No	Interactions	Mode of Action	Description
1.	$TNF \rightarrow TNFR1_a$	Binding reaction	Binding of TNFα ligand with its receptor-1 [26]
2.	$TNFR1a \rightarrow RAF$	Enzymatic reaction	Raf complex gets activated via Phosphorylation at S338 and Y341. [1,27]
3.	$TNFR1a \rightarrow MKK$	Enzymatic reaction	TNFR1a mediated activation of MKK4 and MKK7 [1]
4.	$TNFR1a \rightarrow NF\kappa B$	Enzymatic reaction	Activation of dimeric trancription factors [10]
5.	$TNFR1a \rightarrow CER$	Catalysis reaction	Ceramide formation via activation of sphingomyelinases [2]
6.	$TNFR1a \rightarrow PI3K$	Enzymatic reaction	Activation of PI3K by TNFα [16]
7.	$RAF \rightarrow ERK$	Enzymatic reaction	RAF-MEK-ERK is a RAS activated protein kinase cascade [1].
8.	$ERK \dashv RAF$	Enzymatic reaction	ERK induced phosphorylation of B-RAF on T753 promotes the disassembly of RAF heterodimer. [28]

9.	$MKK \rightarrow JNK$	Enzymatic reaction	MKK4 and MKK7 activates JNK by phosphorylating on Thr183 and Tyr185 residue [1].
10.	$JNK \rightarrow ROS$	Production reaction	pJNK mediated ROS production [5].
11.	$ROS \rightarrow MKK$	Enzymatic reaction	Increased level of ROS activates ASK1 which in turn activates MKK4 and MKK7 [29].
12.	$JNK \rightarrow Caspase3$	Catalytic and enzymatic reaction	JNK activates Caspase3 through a jBid-SMAC dependent mechanism [8].
13.	$JNK \rightarrow AKT$	Enzymatic reaction	Activation of AKT by pJNK through PDK-1 [25].
14.	$JNK \rightarrow ERK$	Enzymatic reaction	JNK indirectly activates ERK through multiple interactive pathway
15.	$ERK \dashv JNK$	Enzymatic reaction	pERK inhibits pJNK [6].
16.	$NF\kappa B \rightarrow XG$	Transcriptional activation	NFκB transcription factor induces transcription of anti-apoptotic proteins like XIAP and Gadd45B [10].
17.	$JNK \rightarrow NF\kappa B$	Enzymatic reaction	JNK indirectly activates $NF\kappa B$ through multiple interactive pathway
18.	$XG \dashv JNK$	Enzymatic reaction	pJNK activation is inhibited by NFκB regulated XIAP and Gadd45B [13].
19.	$PTEN \dashv NF\kappa B$	Enzymatic reaction	PTEN phosphatase inhibits NFκB activity [19].
20.	$NF\kappa B \dashv PTEN$	Transcriptional reaction	NFκB inhibits PTEN [15].
21.	$PTEN \dashv PI3K$	Enzymatic reaction	PTEN, a tumor supressor inhibits PI3K function [14].

22.	$NFkB \dashv Bcl2$	Transcriptional reaction	NFκB represses Bcl2 transcription [17].
23.	$NFkB \dashv Caspase3$	Multiple Inhibition	NFκB transcription factor induces transcription of various anti-apoptotic proteins [12].
24.	$ERK \dashv Caspase3$	Enzymatic reaction	pERK inhibits Caspase3 activation via phosphorylation of Caspase9 [7].
25.	$AKT \dashv Caspase3$	Enzymatic reaction	pAKT inhibit cellular apoptosis by phosphorylating pro-Caspase9 at Ser 196 [22].

S2.3: Description of the phenomenological terms used in the model equations

Phenomenological terms included in the ODE model (S1 Table) are discussed in this section. Biochemical reaction rate (Eq. 5 in S1 Table) corresponding to inhibition of pJNK by pERK is given by

$$-\left(\frac{K_{eij}\times pERK\times pJNK}{K_{eij1}+K_{eij2}\times pERK}\right) \qquad [S2.3.1]$$

which allows introduction of a threshold activation due to pERK mediated inhibition of pJNK. We capture the rate (Eq. 6 in S1 Table) corresponding to inhibitory effect of TPL on basal NFκB and also activation by TNFR1a by

$$\frac{(K_{bnf}\times NFkB)}{(1+K_{inh}\times TPL)} \qquad [S2.3.2]$$

and

$$\frac{(K_{tnf}\times NFkB\times TNFR1a)}{(1+K_{inh}\times TPL)}, \qquad [S2.3.3]$$

respectively. The effect of pJNK, pERK and pAKT, respectively on Caspase3 has been introduced into the corresponding model equation (Eq. 16 in S1 Table) by

$$\frac{(K_{jacs3}\times Cs3\times pJNK)}{(K_{jac2}+(n1\times pJNK))}, \qquad [S2.3.4]$$

$$-\frac{(K_{eics3}\times Cs3a\times pERK)}{(K_{eic1}+(K_{eic2}\times pERK))} \qquad [S2.3.5]$$

and

$$-\frac{(K_{aics3}\times Cs3a\times pAKT^{Kn2})}{(K_{aic1}+(K_{aic2}\times pAKT^{Kn2}))}, \qquad [S2.3.6]$$

respectively. Note that the phenomenological rate expression in Eq. S2.3.4 permits threshold activation of Caspase3 by pJNK. On the other hand, Eqs S2.3.5 and S2.3.6 incorporate repression by pERK and pAKT, respectively on Caspase3 beyond a certain threshold.

S2.4: Identifiability of the estimated parameters

The objective function (Eq 5, Method M6, Main text) was minimized to estimate the parameters based on the experimental training dataset using the methodology schematically depicted in S3 Fig. The optimization was performed using PottersWheel software (version 4.1.1) [30] wherein we chose the "Trustregion" method.

We considered 2000 fit sequences by generating randomly the starting guess parameter set. We chose 3% best fit out of these 2000 sequences that satisfies the fitting criteria based on χ^2 values. The boxplot in S4 Fig demonstrates the identifiability of these 3% best fit parameter sets. Almost 80% of the parameters are well constrained and the relative uncertainly in identifying is below 2.5%, which signifies a narrowed distribution of the parameter sets for a multi-experimental fitting.

S2.5: Model trajectories for the best-fit parameter set

Out of the 3% best fit parameters, the model trajectories for the one with the lowest χ^2 of 89.21 and Akaike Information Criterion of 488.3 is shown in S5 Fig for all three stimulation conditions along with the corresponding experimentally measured dynamics.

References

1. Dhillon AS, Hagan S, Rath O, Kolch W. MAP kinase signalling pathways in cancer. Oncogene. 2007;26: 3279–3290. doi: 10.1038/sj.onc.1210421. PMID: 17496922.

2. Sawada M, Kiyono T, Nakashima S, Shinoda J, Naganawa T, Hara S, et al. Molecular mechanisms of TNF-α-induced ceramide formation in human glioma cells: p53-mediated oxidant stress-dependent and-independent pathways. Cell Death \& Differ. 2004;11: 997–1008. https://doi.org/10.1038/sj.cdd.4401438

3. Gangoiti P, Granado MH, Wang SW, Kong JY, Steinbrecher UP, Gómez-Muñoz A. Ceramide 1-phosphate stimulates macrophage proliferation through activation of the PI3-kinase/PKB, JNK and ERK1/2 pathways. Cell Signal. 2008;20: 726–736. doi: 10.1016/j.cellsig.2007.12.008. Epub 2007 Dec 17. PMID: 18234473.

4. Grummisch JA, Jadavji NM, Smith PD. The pleiotropic effects of tissue plasminogen activator in the brain: implications for stroke recovery. Neural Regen Res. 2016;11: 1401. https://doi.org/10.4103/1673-5374.191204. PMID: 27857733; PMCID: PMC5090832.

5. Ventura J-J, Cogswell P, Flavell RA, Baldwin AS, Davis RJ. JNK potentiates TNF-stimulated necrosis by increasing the production of cytotoxic reactive oxygen species. Genes \& Dev. 2004;18: 2905–2915. doi: 10.1101/gad.1223004. Epub 2004 Nov 15. PMID: 15545623; PMCID: PMC534651.

6. Monick MM, Powers LS, Gross TJ, Flaherty DM, Barrett CW, Hunninghake GW. Active ERK contributes to protein translation by preventing JNK-dependent inhibition of protein phosphatase 1. J Immunol. 2006;177: 1636–1645. doi: 10.4049/jimmunol.177.3.1636. PMID: 16849472.

7. Allan LA, Morrice N, Brady S, Magee G, Pathak S, Clarke PR. Inhibition of caspase-9 through phosphorylation at Thr 125 by ERK MAPK. Nat Cell Biol. 2003;5: 647–654. https://doi.org/10.1128/MCB.25.23.10543-10555.2005 PMID: 16287866; PMCID: PMC1291226.

8. Deng Y, Ren X, Yang L, Lin Y, Wu X. A JNK-dependent pathway is required for TNFα-induced apoptosis. Cell. 2003;115: 61–70. doi: 10.1016/s0092-8674(03)00757-8. PMID: 14532003.

9. Lamb JA, Ventura J-J, Hess P, Flavell RA, Davis RJ. JunD mediates survival signaling by the JNK signal transduction pathway. Mol Cell. 2003;11: 1479–1489. doi: 10.1016/s1097-2765(03)00203-x. PMID: 12820962.

10. Karin M. Lin A.: NF-KB at the crossroads of life and death. Nat Immunol. 2002;3: 221–227. doi: 10.1038/ni0302-221. PMID: 11875461.

11. Lee KY, Chang W, Qiu D, Kao PN, Rosen GD. PG490 (triptolide) cooperates with tumor necrosis factor-α to induce apoptosis in tumor cells. J Biol Chem. 1999;274: 13451–13455. doi: 10.1074/jbc.274.19.13451. PMID: 10224110.

12. Barkett M, Gilmore TD. Control of apoptosis by Rel/NF-$κ$B transcription factors. Oncogene. 1999;18: 6910–6924. doi: 10.1038/sj.onc.1203238. PMID:

10602466.

13. Papa S, Zazzeroni F, Pham CG, Bubici C, Franzoso G. Linking JNK signaling to NF-κB: a key to survival. J Cell Sci. 2004;117: 5197–5208. doi: 10.1242/jcs.01483. PMID: 15483317.

14. Carracedo A, Pandolfi PP. The PTEN--PI3K pathway: of feedbacks and cross-talks. Oncogene. 2008;27: 5527–5541. https://doi.org/10.1038/onc.2008.247

15. Vasudevan KM, Gurumurthy S, Rangnekar VM. Suppression of PTEN expression by NF-κB prevents apoptosis. Mol Cell Biol. 2004;24: 1007–1021. doi: 10.1128/MCB.24.3.1007-1021.2004. PMID: 14729949; PMCID: PMC321419.

16. Ozes ON, Akca H, Gustin JA, et al. Tumor Necrosis Factor-α/Receptor Signaling Through the Akt Kinase. In *Cell Signaling in Vascular Inflammation*, pages 13–22. Springer, 2005.

17. Sohur US, Dixit MN, Chen C-L, Byrom MW, Kerr LD. Rel/NF-κB Represses bcl-2 Transcription in pro-B Lymphocytes. Gene Expr J Liver Res. 1999;8: 219–229. PMID: 10794524; PMCID: PMC6157363.

18. Mortenson MM, Galante JG, Gilad O, Schlieman MG, Virudachalam S, Kung H-J, et al. BCL-2 functions as an activator of the AKT signaling pathway in pancreatic cancer. J Cell Biochem. 2007;102: 1171–1179. doi: 10.1002/jcb.21343. PMID: 17960583.

19. Gustin JA, Maehama T, Dixon JE, Donner DB. The PTEN tumor suppressor protein inhibits tumor necrosis factor-induced nuclear factor $κ$B activity. J Biol Chem. 2001;276: 27740–27744. doi: 10.1074/jbc.M102559200. Epub 2001 May 16. PMID: 11356844.

20. Salinas M, López-Valdaliso R, Mart\ín D, Alvarez A, Cuadrado A. Inhibition of PKB/Akt1 by C2-ceramide involves activation of ceramide-activated protein phosphatase in PC12 cells. Mol Cell Neurosci. 2000;15: 156–169. doi: 10.1006/mcne.1999.0813. PMID: 10673324.

21. Dobrowsky RT, Kamibayashi C, Mumby MC, Hannun YA. Ceramide activates heterotrimeric protein phosphatase 2A. J Biol Chem. 1993;268: 15523–15530. PMID: 8393446.

22. Cardone MH, Roy N, Stennicke HR, Salvesen GS, Franke TF, Stanbridge E, et al. Regulation of cell death protease caspase-9 by phosphorylation. Science 80. 1998;282: 1318–1321. doi: 10.1126/science.282.5392.1318. PMID: 9812896.

23. Rommel C, Clarke BA, Zimmermann S, Nuñez L, Rossman R, Reid K, et al. Differentiation stage-specific inhibition of the Raf-MEK-ERK pathway by Akt. Science 80. 1999;286: 1738–1741. doi: 10.1126/science.286.5445.1738. PMID: 10576741.

24. Park H-S, Kim M-S, Huh S-H, Park J, Chung J, Kang SS, et al. Akt (protein kinase B) negatively regulates SEK1 by means of protein phosphorylation. J Biol Chem. 2002;277: 2573–2578. doi: 10.1074/jbc.M110299200. Epub 2001 Nov 13. PMID: 11707464.

25. Shaw J, Kirshenbaum LA. Prime time for JNK-mediated Akt reactivation in hypoxia-reoxygenation. Am Heart Assoc; 2006.

https://doi.org/10.1161/01.RES.0000200397.22663.b6

26. Wajant H, Pfizenmaier K, Scheurich P. Tumor necrosis factor signaling. Cell Death \& Differ. 2003;10: 45–65. doi: 10.1038/sj.cdd.4401189. PMID: 12655295.

27. Mason CS, Springer CJ, Cooper RG, Superti-Furga G, Marshall CJ, Marais R. Serine and tyrosine phosphorylations cooperate in Raf-1, but not B-Raf activation. EMBO J. 1999;18: 2137–2148. doi: 10.1093/emboj/18.8.2137. PMID: 10205168; PMCID: PMC1171298.

28. Rushworth LK, Hindley AD, O'Neill E, Kolch W. Regulation and role of Raf-1/B-Raf heterodimerization. Mol Cell Biol. 2006;26: 2262–2272. doi: 10.1128/MCB.26.6.2262-2272.2006. PMID: 16508002; PMCID: PMC1430271.

29. Soga M, Matsuzawa A, Ichijo H. Oxidative stress-induced diseases via the ASK1 signaling pathway. Int J Cell Biol. 2012;2012. https://doi.org/10.1155/2012/439587

30. Maiwald T, Timmer J. Dynamical modeling and multi-experiment fitting with PottersWheel. Bioinformatics. 2008;24: 2037–2043. doi: 10.1093/bioinformatics/btn350. Epub 2008 Jul 9. PMID: 18614583; PMCID: PMC2530888.
