# Supplementary material for: Modulation of signaling cross-talk between pJNK and pAKT generates optimal apoptotic response
Source: PLoS Comput Biol. 2022 Oct 14;18(10):e1010626. doi: 10.1371/journal.pcbi.1010626 (PMC9604984; doi:10.1371/journal.pcbi.1010626)
Supplement: S3 Text — (PDF) [file pcbi.1010626.s003.pdf]

Modulation of signaling cross-talk between pJNK and pAKT generates optimal apoptotic response

Sharmila Biswas[1,¶] , Baishakhi Tikader[2,¶], Sandip Kar[2*], Ganesh A Viswanathan[1*]

[1]Department of Chemical Engineering, Indian Institute of Technology Bombay, Mumbai, India.

[2]Department of Chemistry, Indian Institute of Technology Bombay, Mumbai, India.

¶These authors contributed equally to this work

*Corresponding authors

E-mail: sandipkar@iitb.ac.in, ganeshav@iitb.ac.in

S3 Text

Prediction and sensitivity analysis of transient dynamics of different entities from the proposed model

S3.1: Sensitivity analysis of the model due to TNFα strength and the other model parameters

We performed the model simulations at different TNFα doses. S6 Fig shows that the qualitative trend of the three signaling entities is similar for wide range of TNFα dose (up to 166 ng/ml) and under different TPL concentrations. Note that TNFα stimulation 1000 ng/ml and beyond leads to a perceptible change in the dynamics as compared to those for lower concentrations.

S3.2: Model simulated NFκB dynamics under different conditions

We examined the model predicted NFκB levels under TNFα, TPL and TNFα+TPL conditions. In the model, we assumed that TPL inhibits both the basal levels and the TNFR1 mediated activation of NFκB. In S7 Fig, we show the nature of NFκB dynamical response to these three stimulation conditions. Model simulation predicts that inhibition by TPL significantly reduces the active NFkB fold change in relatively short span of time (S7 Fig, red). This reduction due to TPL could not be restored even in the presence of 100 ng/ml of TNFa in the presence of TPL (S7 Fig, black dashed line). However, in the absence of TPL can strongly activate NFκB. This indicates that TPL strongly reduces the pro-survival signaling downstream of NFκB.

S3.3: Prediction of independent experimental dynamics by model simulations

In order to verify the predictive ability of the model, simulations using model equations in S1 Table were performed at a different stimulation condition TNFα (100 ng/ml) + TPL (10nM). The levels of the three marker proteins were measured on U937 cells under this stimulation conditions (Methods, Main text). The model trajectory predictions and the experimentally measured dynamics are contrasted in S8 Fig. $RMSD$ values were estimated using the expression

$$RMSD = \frac{1}{n}\sum(y_i - \hat{y}_i)^2$$

where, y_i and $\hat{y}_i$, respectively represent experimental and simulated levels. n is the total number of data points.

S3.3: Sensitivity analysis of the model due to TNFα strength and the other model parameters

In S9 Fig, we show the $\log(\Phi_k/\Phi_{wt})$ for pJNK, pAKT and Caspase3 transients capturing sensitivity with respect to the cross-talk parameters (Table I). Φ_{wt} represents the area under the curve (AUC) of the trajectories of pAKT, pJNK and Caspase3 of the best fitted parameter sets. The values corresponding to the best fit set of the cross-talk parameters are specified in Table I. Φ_k represents the AUC of the trajectories of three proteins when the value of a certain cross-talk parameter was increased by 20% as that the best fitted parameter sets. Sensitivity analysis reveals that the parameter K_{naxp1} associated with XG and C1P (S9 Fig-A) significantly affects the pJNK, pAKT and Caspase3 dynamics. The parameter K_{paak} associated with PI3K and pAKT activation (S9 Fig-B) control Caspase3 suggesting strong negative influence of pAKT on Caspase3 levels. All the kinetic parameters associated with Caspase3 (S9 Fig-C and S9 Fig-D) suggest that the parameters were only sensitive for Caspase3 as the downstream Caspase3 activation does not influence the upstream proteins like pAKT and pJNK. Modulating parameters K_{xij} and K_{m4aj} associated with pJNK (S9 Fig-B) affects transients of all three proteins suggesting that pJNK majorly controlled both pAKT and Caspase3 levels.

Table I. Value of the kinetic parameters related to cross-talk

Symbol	Value of the best-fitted parametric sets	20% increased
K_{tnf1}	0.0337	0.04044
K_{tnf2}	0.00015	0.00018
K_{cac1}	0.00341	0.00409
K_{naxp1}	0.0183	0.02196
K_{xij}	0.602	0.7224
K_{cajk}	0.102	0.1224
K_{m4aj}	0.0253	0.03036
K_{eij}	0.0285	0.0342
K_{eij1}	1.36	1.632
K_{eij2}	0.0895	0.10068
K_{tnf}	0.0015	0.0018

K_{jan}	0.00011	0.0001324
K_{pin}	0.0486	0.05832
K_{nip}	0.0996	0.11952
K_{tpi1}	0.0144	0.017328
K_{pip3}	1.56	1.872
K_{paak}	0.259	0.3108
K_{jaa}	0.998	1.1979
K_{tnar}	0.000283	0.0003396
K_{eir}	0.0069	0.00828
K_{air}	2.38	2.856
K_{jar}	0.0735	0.0882
K_{mae}	0.0018	0.00216
K_{paer}	0.00063	0.000756
K_{jae}	0.0104	0.01248
K_{jacs3}	0.0113	0.01356
K_{tcs}	0.04316	0.05179
K_{nics3}	0.577	0.6924
K_{aics3}	0.00378	0.004536
K_{eics3}	0.00021	0.000252
K_{eic1}	0.7635	0.9162
K_{eic2}	4.62	5.544
K_{tkk1}	0.0768	0.09216
K_{rk4}	0.1	0.12
K_{aik4}	0.005	0.006
$n1$	0.0186	0.02232
K_{aic1}	0.0077	0.00924
K_{aic2}	0.00076	0.000912
K_{tcr}	0.00665	0.00798
K_{picr}	0.108	0.1296
K_{acp}	0.069	0.0828
K_{cpia}	1.09	1.308

K_{nix}	11.36	13.632
K_{xaa}	0.494	0.5928
