# Supplementary material for: Modulation of signaling cross-talk between pJNK and pAKT generates optimal apoptotic response
Source: PLoS Comput Biol. 2022 Oct 14;18(10):e1010626. doi: 10.1371/journal.pcbi.1010626 (PMC9604984; doi:10.1371/journal.pcbi.1010626)
Supplement: S4 Text — (PDF) [file pcbi.1010626.s004.pdf]

Modulation of signaling cross-talk between pJNK and pAKT generates optimal apoptotic response

Sharmila Biswas[1,¶] , Baishakhi Tikader[2, ¶], Sandip Kar[2*], Ganesh A Viswanathan[1*]

[1]Department of Chemical Engineering, Indian Institute of Technology Bombay, Mumbai, India.

[2]Department of Chemistry, Indian Institute of Technology Bombay, Mumbai, India.

[¶]These authors contributed equally to this work

[*]Corresponding authors

E-mail: sandipkar@iitb.ac.in, ganeshav@iitb.ac.in

S4 Text

Reaction flux analysis of the three marker proteins

The reaction flux analysis was performed to evaluate the contribution of different entities in the TNFα network (Fig 2A, main text, and S2 Fig) in modulating pAKT, pJNK and Caspase3 dynamics. S4 Table presents the expression of fluxes contributing to the dynamics of pJNK, NFκB, pAKT, and Caspase3. For the case of pJNK, fluxes J_i $\forall i = 1{,}6$ quantify the rate of each of the terms contributing its dynamics (Eq. 5 in S1 Table). Similarly, N_i, A_i and C_i, respectively capture the fluxes of the terms contributing to the dynamics of NFκB, pAKT and Caspase3, respectively.

The evolution of the fluxes corresponding to pJNK, pAKT, and Caspase3 are shown in S10 Fig for the three stimulation conditions. On the other hand, those for NFκB are shown in Fig I.

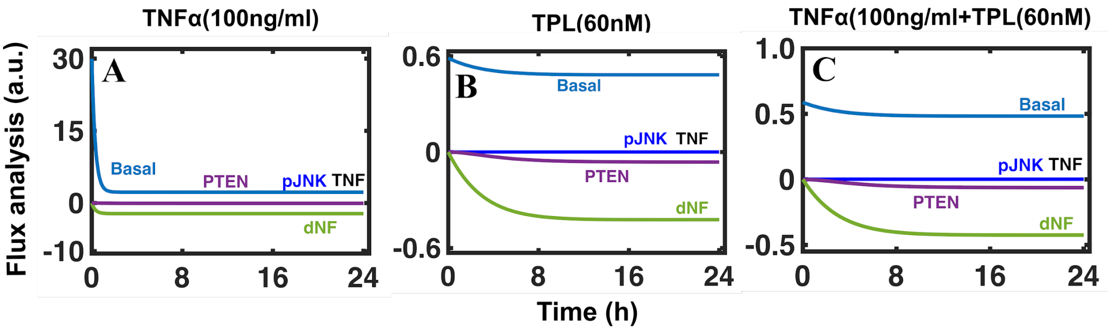

Fig I. Temporal evolution of fluxes contributing to the dynamics of NFκB under the three stimulation conditions. Expression for these fluxes are provided in S4 Table.
