# Supplementary material for: Modulation of signaling cross-talk between pJNK and pAKT generates optimal apoptotic response
Source: PLoS Comput Biol. 2022 Oct 14;18(10):e1010626. doi: 10.1371/journal.pcbi.1010626 (PMC9604984; doi:10.1371/journal.pcbi.1010626)
Supplement: S5 Text — (PDF) [file pcbi.1010626.s005.pdf]

Modulation of signaling cross-talk between pJNK and pAKT generates optimal apoptotic response

Sharmila Biswas[1,¶] , Baishakhi Tikader[2, ¶], Sandip Kar[2*], Ganesh A Viswanathan[1*]

[1]Department of Chemical Engineering, Indian Institute of Technology Bombay, Mumbai, India.

[2]Department of Chemistry, Indian Institute of Technology Bombay, Mumbai, India.

[¶]These authors contributed equally to this work

[*]Corresponding authors

E-mail: sandipkar@iitb.ac.in, ganeshav@iitb.ac.in

S5 Text

Branch analysis quantifying the synergistic dynamic cross-talk signaling

Comprehensive list of branches originating from TNFR1 or NFκB and ending in pAKT or pJNK are presented in S5 Table. Major branches culled out from this list are presented in Table 1, main text. Using Eq. 3, main text, the time-dependent synergism due to the branches listed in S5 Table is presented in S11 Fig.
