# Supplementary material for: Modulation of signaling cross-talk between pJNK and pAKT generates optimal apoptotic response
Source: PLoS Comput Biol. 2022 Oct 14;18(10):e1010626. doi: 10.1371/journal.pcbi.1010626 (PMC9604984; doi:10.1371/journal.pcbi.1010626)
Supplement: S6 Text — (PDF) [file pcbi.1010626.s006.pdf]

Modulation of signaling cross-talk between pJNK and pAKT generates optimal apoptotic response

Sharmila Biswas[1,¶] , Baishakhi Tikader[2, ¶], Sandip Kar[2*], Ganesh A Viswanathan[1*]

[1]Department of Chemical Engineering, Indian Institute of Technology Bombay, Mumbai, India.

[2]Department of Chemistry, Indian Institute of Technology Bombay, Mumbai, India.

[¶]These authors contributed equally to this work

[*]Corresponding authors

E-mail: sandipkar@iitb.ac.in, ganeshav@iitb.ac.in

S6 Text

Model analysis under inhibitory conditions: Wortmannin and SP600125

S6.1: Wortmannin (Wort) and SP600125 (SP6) Inhibitory model of TNFα network

In order to capture the effect of Wort inhibitor on MKK mediated JNK phosphorylation, PI3K mediated AKT phosphorylation and NFκB mediated XG phosphorylation, we modified Eqs. 3, 5 and 9 in S1 Table by introducing an inhibitory action in a phenomenological manner. These modified equations are

$$\frac{dpJNK}{dt} = (K_{bjnk} \times JNK) + (K_{cajk} \times JNK \times C1P_a) - \left(\frac{K_{eij} \times pERK \times pJNK}{K_{eij1} + K_{eij2} \times pERK}\right) - (K_{djnk} \times pJNK) + \left(\frac{K_{m4aj} \times JNK \times MKK_a}{1 + (K_{kim} \times Wort)}\right) - (K_{xij} \times XG_a \times pJNK) \quad [S6.1.1]$$

$$\frac{dpAKT}{dt} = \left((K_{bak} \times AKT) + \left(\frac{K_{paak} \times PI3K_a \times AKT}{1 + (K_{iak} \times Wort)}\right) - (K_{bdak} \times pAKT) + (K_{jaa} \times pJNK \times AKT) - (K_{cpia} \times CAPP_a \times pAKT) + (K_{xaa} \times Bcl2_a \times AKT)\right) \quad [S6.1.2]$$

$$\frac{dXG_a}{dt} = \left((K_{bxg} \times XG) + \left(\frac{K_{naxp1} \times XG \times NFkB_a}{1 + (K_{kxg} \times Wort)}\right) - ((K_{dxg} \times XG_a))\right) \quad [S6.1.3]$$

Similarly, Eqs 5 and 9 in S1 Table were modified to incorporate the inhibitory action of SP6 on MKK mediated JNK phosphorylation and PI3K mediated AKT phosphorylation. These modified equations are

$$\frac{dpJNK}{dt} = (K_{bjnk} \times JNK) + (K_{cajk} \times JNK \times C1P_a) - \left(\frac{K_{eij} \times pERK \times pJNK}{K_{eij1} + K_{eij2} \times pERK}\right) - (K_{djnk} \times pJNK) + \left(\frac{K_{m4aj} \times JNK \times MEKK_a}{1 + (K_{kis} \times SP6)}\right) - (K_{xij} \times XG_a \times pJNK) \quad [S6.1.4]$$

$$\frac{dpAKT}{dt} = \left((K_{bak} \times AKT) + (K_{paak} \times PI3K_a \times AKT) - (K_{bdak} \times pAKT) + (K_{jaa} \times pJNK \times AKT) - (K_{cpia} \times CAPP_a \times pAKT) + \left(\frac{K_{xaa} \times Bcl2_a \times AKT}{1 + (K_{kib} \times SP6)}\right)\right) \quad [S6.1.5]$$

S6.2: Model predicted and experimentally measured transients with and without inhibition

A comparison of the inhibitory model transients with that sans inhibition via Wort is in Fig IA. This is also juxtaposed with a similar comparison for experimental measurements with and without Wort inhibition conditions. Moreover, these comparisons for the case of with and without SP6 inhibition are in Fig IB.

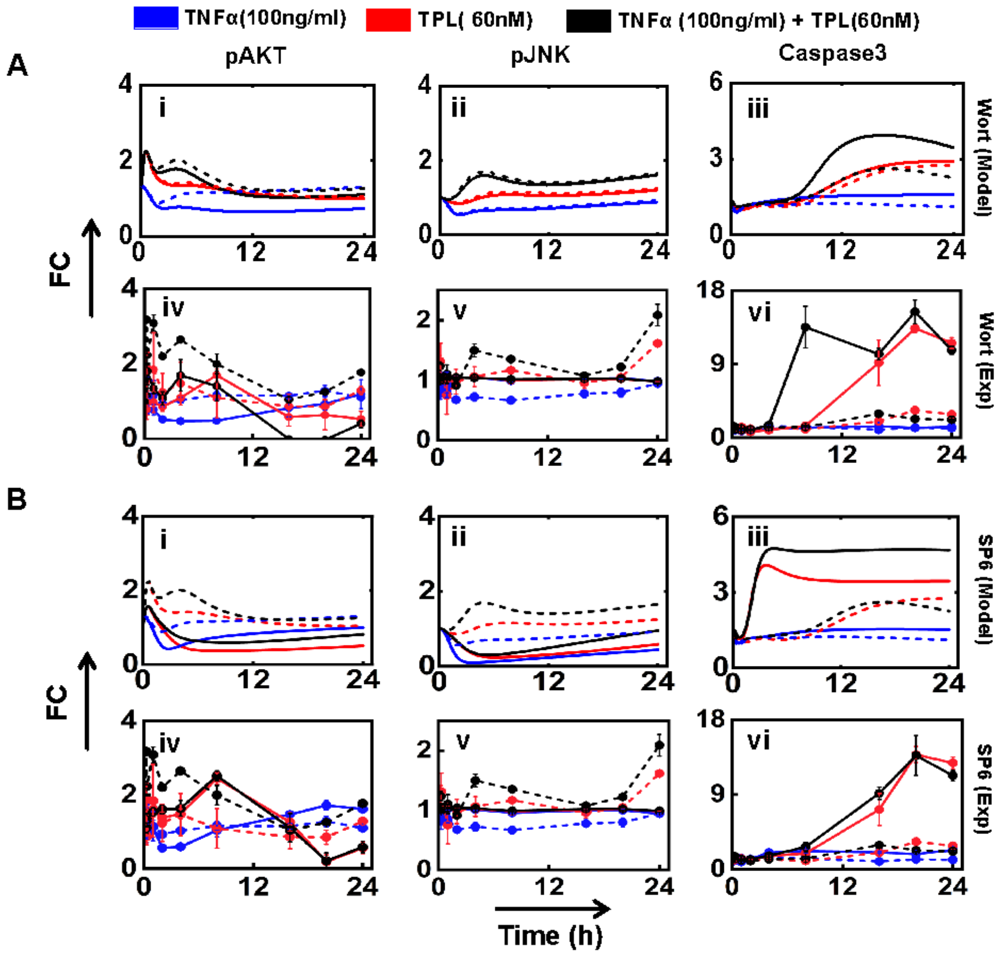

Fig I. Comparison of the model and experimental dynamics of the marker proteins under (A) Wort or (B) SP6 inhibitory treatment for all three stimulation conditions. While solid lines represent inhibitory condition, dashed lines correspond to those without inhibition.

S6.3: Flux analysis for the Wort and SP6 inhibitory models

In order to perform the flux analysis of the inhibitory models, the modified model specified in S6.1 along with other equations provided in S1 Table were employed. For the case of Wort inhibition, fluxes J_5 and A_2 in S4 Table were modified with those rates incorporating its inhibitory action, as specified in Eq. S6.1.1 and Eq. S6.1.2. Besides, the flux corresponding to NFκB activating XG was specified by the second term in the

rhs of Eq. S6.1.3. Similarly, for SP6 inhibition, the inhibitory effects were included in fluxes J_5 and A_6 in S4 Table using the modification specified in Eq. S6.1.4 and S6.1.5.

For all three stimulation conditions, we show a comparison of the evolution of fluxes due to important entities corresponding to the three marker proteins in the presence and absence of Wort (S12 Fig) and SP6 (S13 Fig) inhibition.

The flux analysis for Wort inhibitor (S12 Fig) suggests that, in case of pJNK, the inhibition by Wort was more pronounced in suppressing MKK mediated activation of pJNK. Thus, the effect of the entity MKK is important under all conditions. Flux analysis suggests that even though Wort directly inhibits pAKT activation via PI3K, it has a moderate influence in regulating pAKT transients. Thus, under the action of Wort inhibitor, pJNK is expected to play a crucial role in regulating cross-talk to modulate Caspase3 transients.

Under only TNFα condition, pJNK and PI3K helps to overcome the CAPP facilitated inhibition and control the late activation in the pAKT dynamics in the absence of inhibition. As the retardation effect of Wort on pJNK is comparably less under TNFα stimuli compare to other two conditions (TPL, TNFα+TPL) (S12 Fig-i,iv,vii, blue solid lines), a significant increment has been observed in pAKT dynamics after 12 h when treated with Wort inhibitor (Fig 6Ai, Main text).

The flux study (S12 Fig) also reveals that the early and later phase response of Caspase3 dynamics is intriguingly controlled by NFκB, pAKT, and pJNK in a synchronized manner for the three stimulation conditions (Fig 6Aiii). Under only TNFα condition, as NFκB majorly suppresses Caspase3 activation (S12 Fig-iii), the function of pAKT and pJNK is minimal, resulting in a slight increase in Caspase3 dynamics as compared to no inhibition (Fig 6Aiii, blue line). In absence of Wort, for both only TPL and TNFα+TPL stimulation conditions (S12 Fig-vi,ix, dotted line), the early response of Caspase3 is mostly regulated by pAKT and pJNK. Under Wort inhibition, the pAKT and NFκB negative contributions to Caspase3 significantly reduces after about 5 hours (S12 Fig-vi,ix, solid line), resulting in a rapid rise in Caspase3 dynamics at approximately 5 hours (Fig 6Aiii). Thus, the interplay between the pAKT and pJNK mediated inhibition regulates the dynamic response of Caspase3. The flux analysis performed under SP6 inhibitory conditions (Fig 6Bi,iii and S13 Fig) showed by and large similar responses contributed by different nodes for all conditions as observed

under the treatment of Wort, thereby reproducing nearly similar dynamical response for pAKT, pJNK and Caspase3 in three different experimental conditions.

S6.4: Branch analysis for the Wort inhibitory model

The branch analysis was performed for the inhibitory conditions. The fluxes for interactions that were inhibited by either Wort or SP6 are as those specified in S6.2 Text. In Fig II, we compare the time-dependent synergism with and without Wort inhibitor facilitated by the major branches listed in Table 1, main text. In Fig II, we compare the time-dependent synergism with and without Wort inhibitor facilitated by the major branches listed in Table 1, main text. Similarly, in Fig III, we compare the time-dependent synergism with and without SP6 inhibitor facilitated by the major branches.

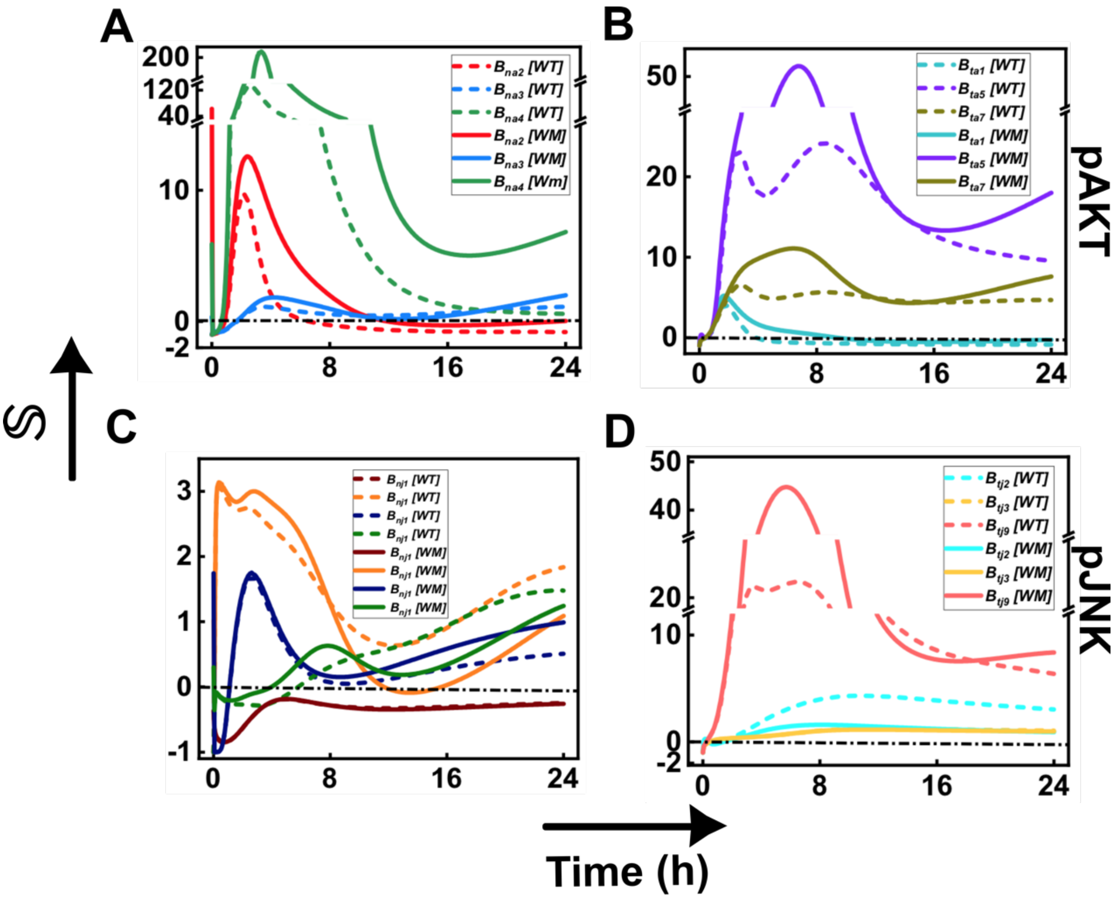

Fig II: Comparison of the time-dependent synergism due of major branches listed in Table 1 (main text) in the presence and absence of Wort inhibitor. The solid and dashed lines, respectively represent the time-dependent synergism in branches from (A) NFκB to pAKT, (B) TNFR1 to pAKT, (C) NFκB to pJNK and (D) TNFR1 to pJNK under presence and absence of Wort inhibition.

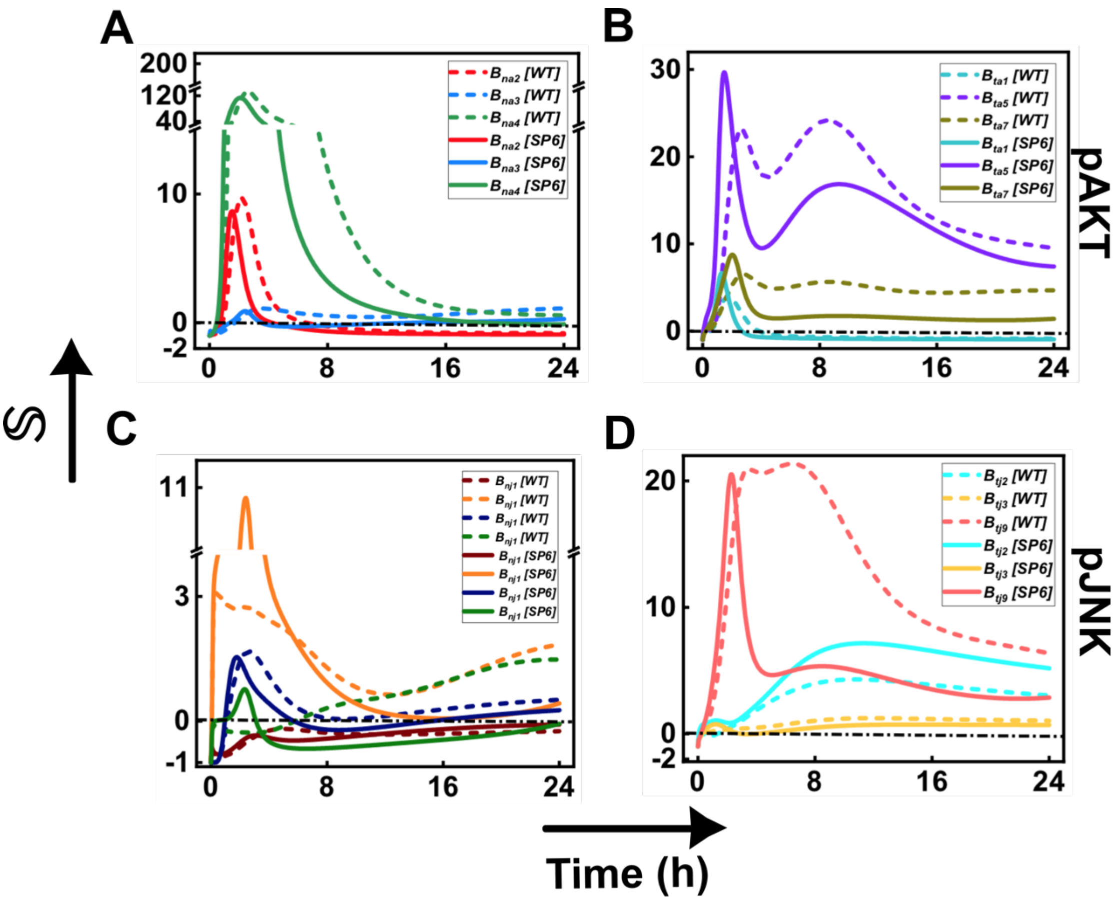

Fig III: Comparison of the time-dependent synergism due of major branches listed in Table 1 (main text) in the presence and absence of SP6 inhibitor. The solid and dashed lines, respectively represent the time-dependent synergism in branches from (A) NFκB to pAKT, (B) TNFR1 to pAKT, (C) NFκB to pJNK and (D) TNFR1 to pJNK under presence and absence of SP6 inhibition.
