# Supplementary material for: Modulation of signaling cross-talk between pJNK and pAKT generates optimal apoptotic response
Source: PLoS Comput Biol. 2022 Oct 14;18(10):e1010626. doi: 10.1371/journal.pcbi.1010626 (PMC9604984; doi:10.1371/journal.pcbi.1010626)
Supplement: S7 Text — (PDF) [file pcbi.1010626.s007.pdf]

Modulation of signaling cross-talk between pJNK and pAKT generates optimal apoptotic response

Sharmila Biswas[1,¶] , Baishakhi Tikader[2,¶], Sandip Kar[2*], Ganesh A Viswanathan[1*]

[1]Department of Chemical Engineering, Indian Institute of Technology Bombay, Mumbai, India.

[2]Department of Chemistry, Indian Institute of Technology Bombay, Mumbai, India.

[¶]These authors contributed equally to this work

[*]Corresponding authors

E-mail: sandipkar@iitb.ac.in, ganeshav@iitb.ac.in

S7 Text

Semi-quantitative relationship between $\langle AUC_{casp3} \rangle$ and Apoptosis levels

The relationship between model predicted $\langle AUC_{casp3} \rangle$ and the apoptosis levels in Fig 5B, main text is quantified using a fourth order polynomial function

$$\% \, Apoptosis = A_0 + A_1\langle AUC_{casp3} \rangle + A_2\langle AUC_{casp3} \rangle^2 + A_3\langle AUC_{casp3} \rangle^3 + A_4\langle AUC_{casp3} \rangle^4 \qquad [S7.1]$$

The coefficients, estimated using Origin software, corresponding to the three stimulation conditions along with R^2 are presented in S6 Table. This was repeated twice, each by using trajectories obtained by simulations with five different best-fit parameters. The coefficients along with the reduced χ^2 estimates for these two cases as well are in S6 Table. The reduced χ^2 values suggest that the fit is good for TNFα treatment case. However, for TPL and TNFα+TPL cases it overfits.
