# Supplementary figures and images for: Modulation of signaling cross-talk between pJNK and pAKT generates optimal apoptotic response
Source: PLoS Comput Biol. 2022 Oct 14;18(10):e1010626. doi: 10.1371/journal.pcbi.1010626 (PMC9604984; doi:10.1371/journal.pcbi.1010626)

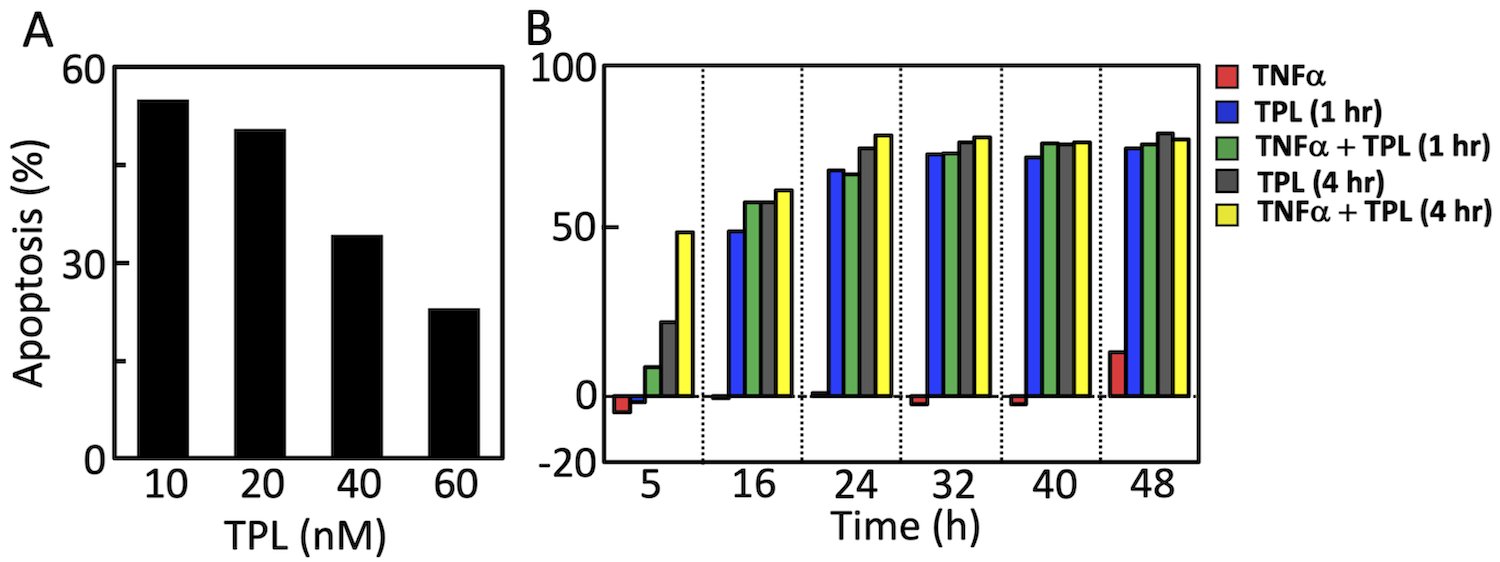

Supplement: S1 Fig — (A) Dose-response of TPL on cell-survival. (B) Effect of TPL pre-treatment duration on apoptosis. Note that negative Apoptosis % in (B) for a few cases are due to negative control (only cells) being more than that when treated. (TIFF) [file pcbi.1010626.s008.tiff]

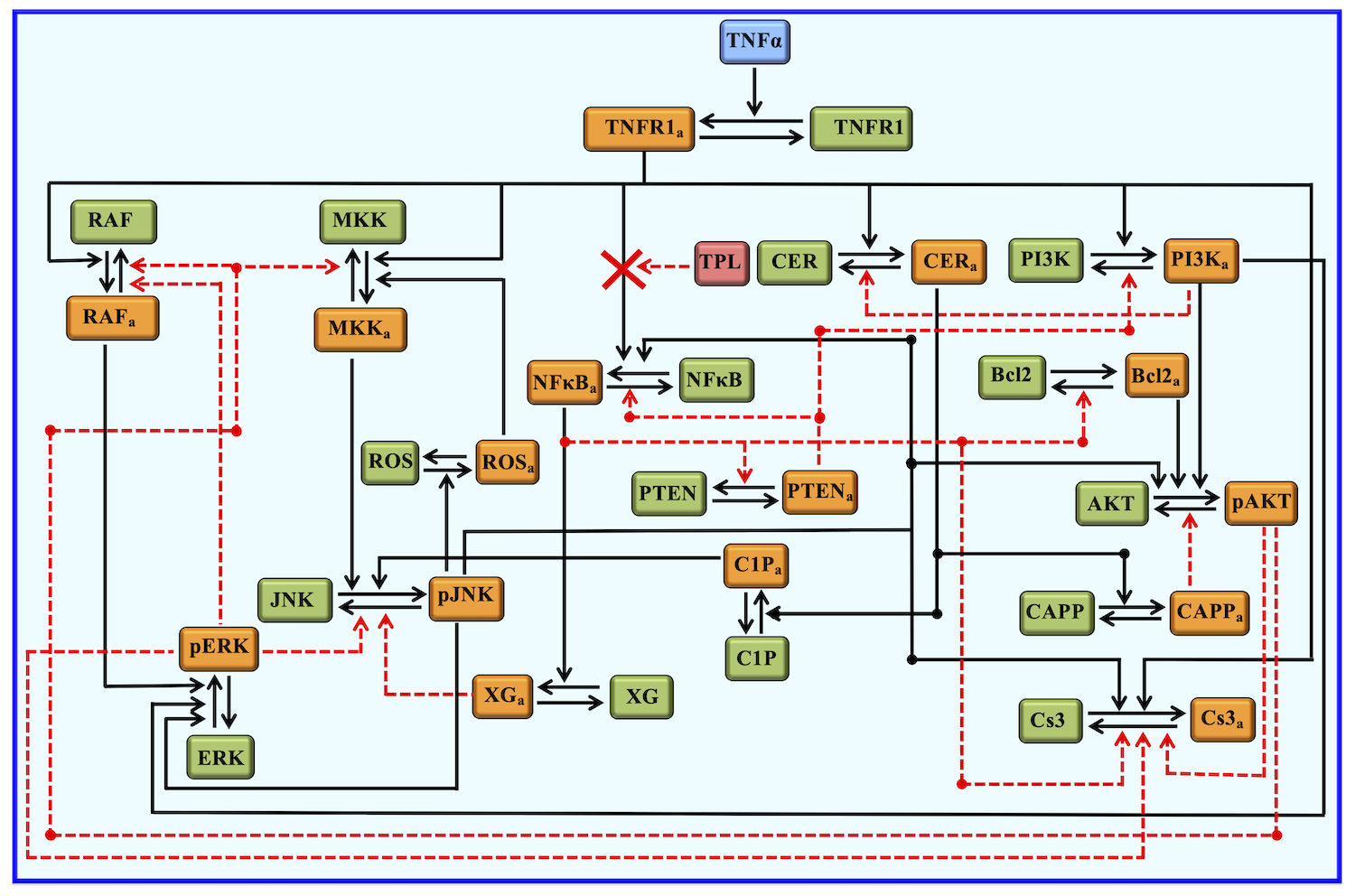

Supplement: S2 Fig — Activation and inhibitory actions are represented by solid (black) and dashed (red) lines, respectively. Green and orange boxes, respectively represent the inactive and active forms of an entity. The model contains 34 species and 81 parameters including 3 scaling constants. (TIFF) [file pcbi.1010626.s009.tiff]

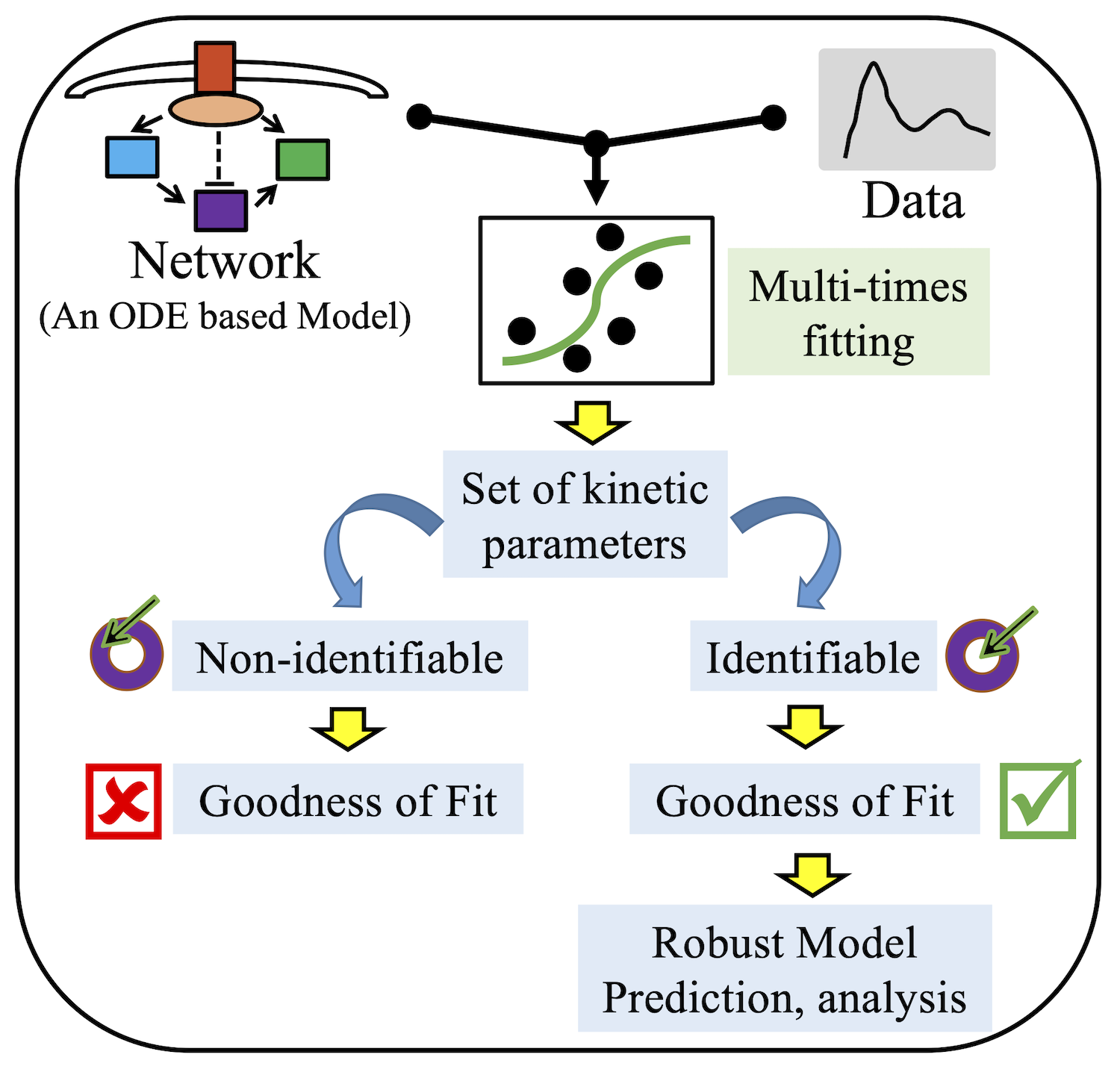

Supplement: S3 Fig — The proposed models were mapped with experimental data and robust model was selected by performing parameter optimization and associated identifiability analysis. (TIFF) [file pcbi.1010626.s010.tiff]

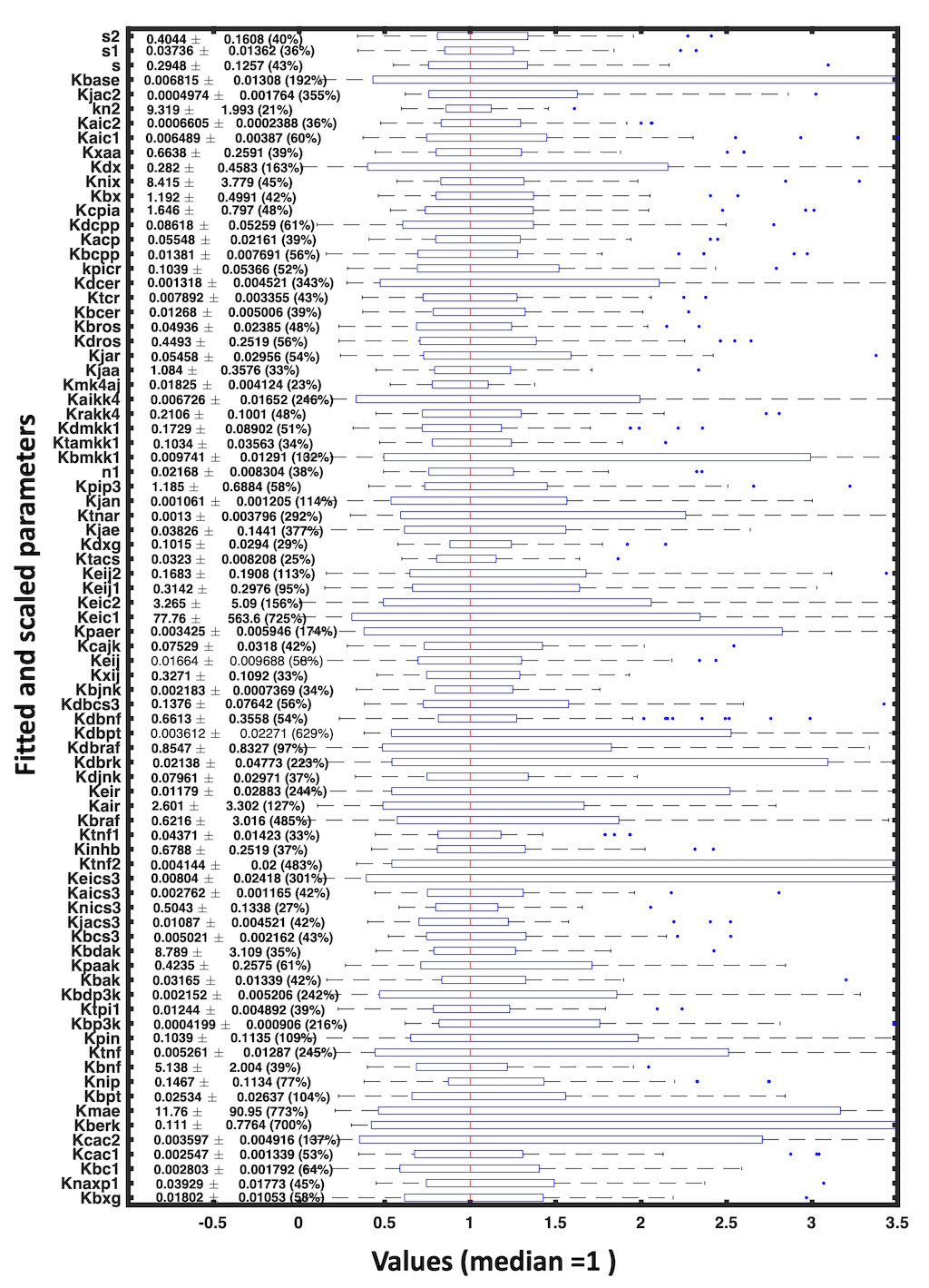

Supplement: S4 Fig — (TIFF) [file pcbi.1010626.s011.tiff]

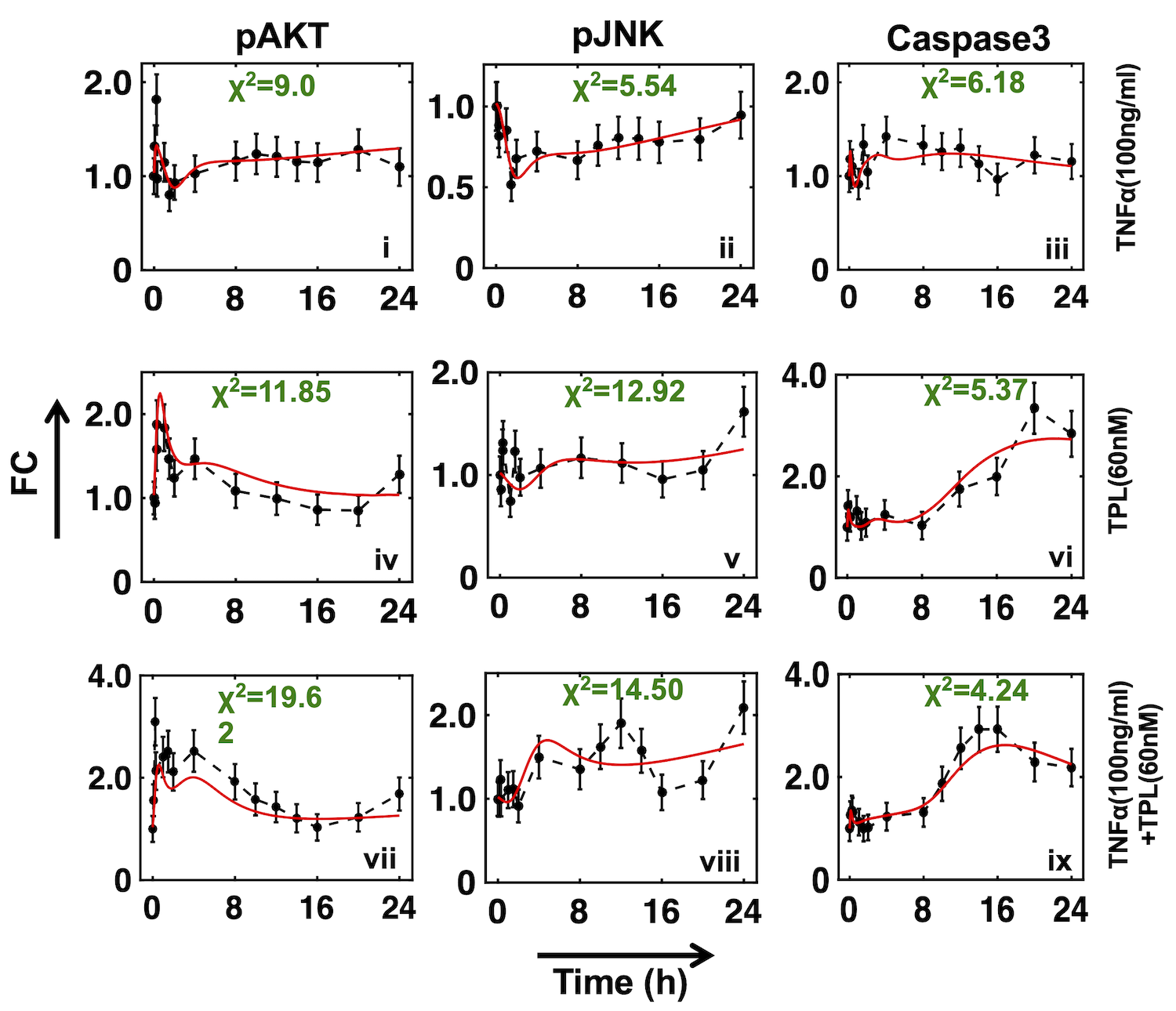

Supplement: S5 Fig — The best-fitted trajectories (red) of ~2000 fits with the experimental FC (circles with appropriate error bars) for pAKT, pJNK and Caspase3 under the three stimulation conditions. The errors are estimated by using a standard error model. (TIFF) [file pcbi.1010626.s012.tiff]

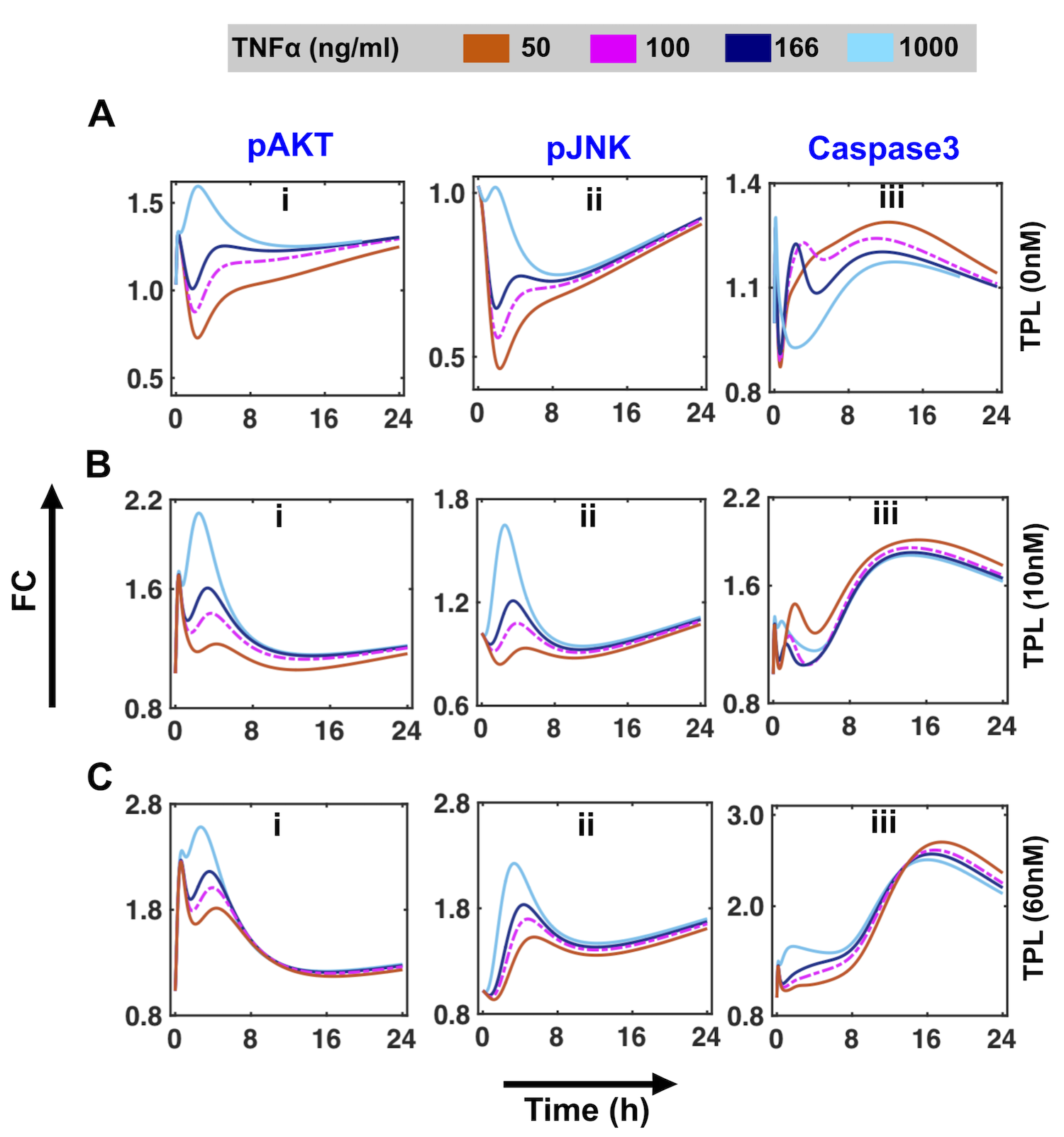

Supplement: S6 Fig — Simulated trajectory of FC by varying TNFα in (A) the absence of TPL, (B) presence of low dose TPL (10nM) and (C) presence of high dose TPL (60nM). (TIFF) [file pcbi.1010626.s013.tiff]

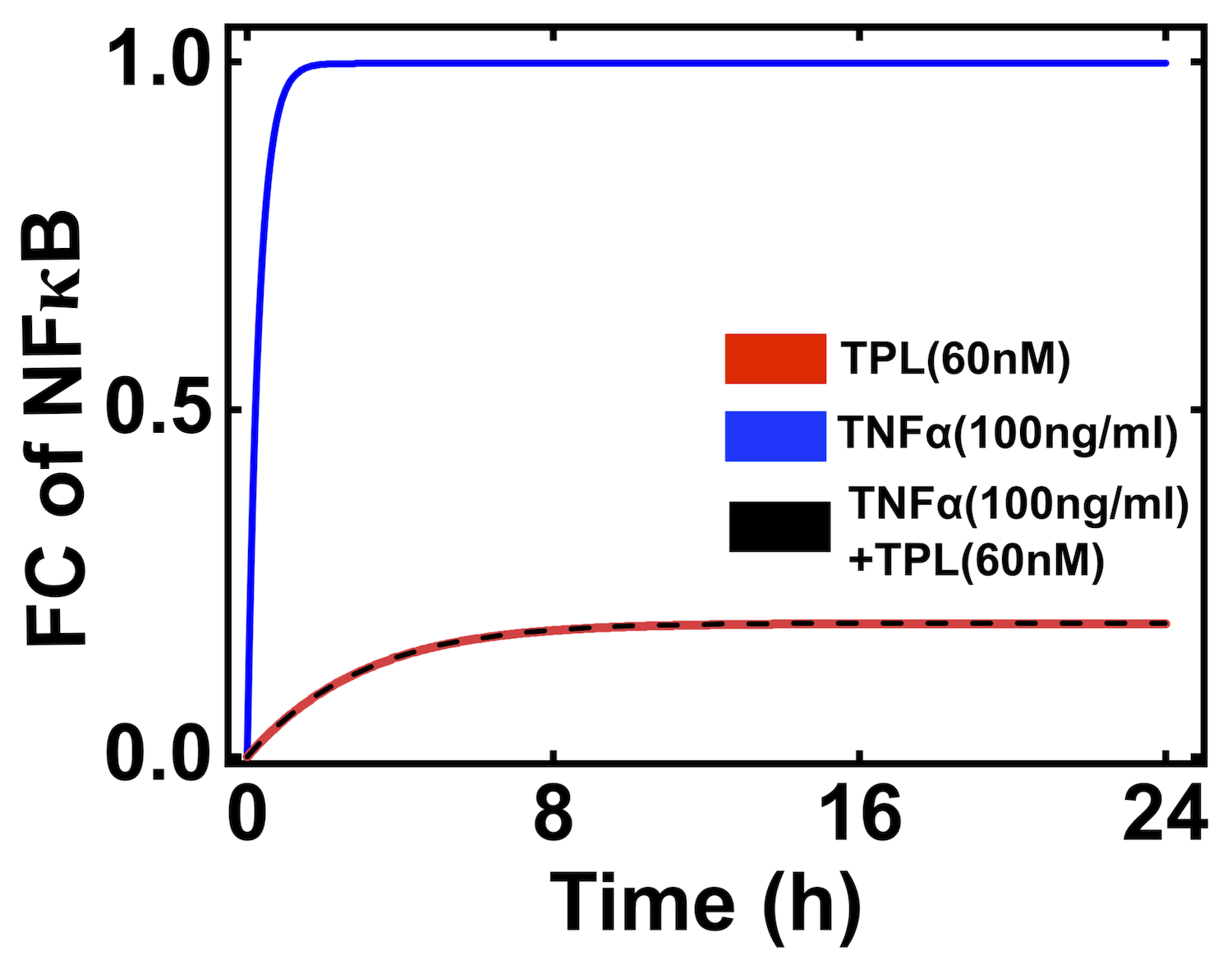

Supplement: S7 Fig — Trajectories of NFκB protein obtained by simulating the model with best-fit parameter set (S4 Table). (TIFF) [file pcbi.1010626.s014.tiff]

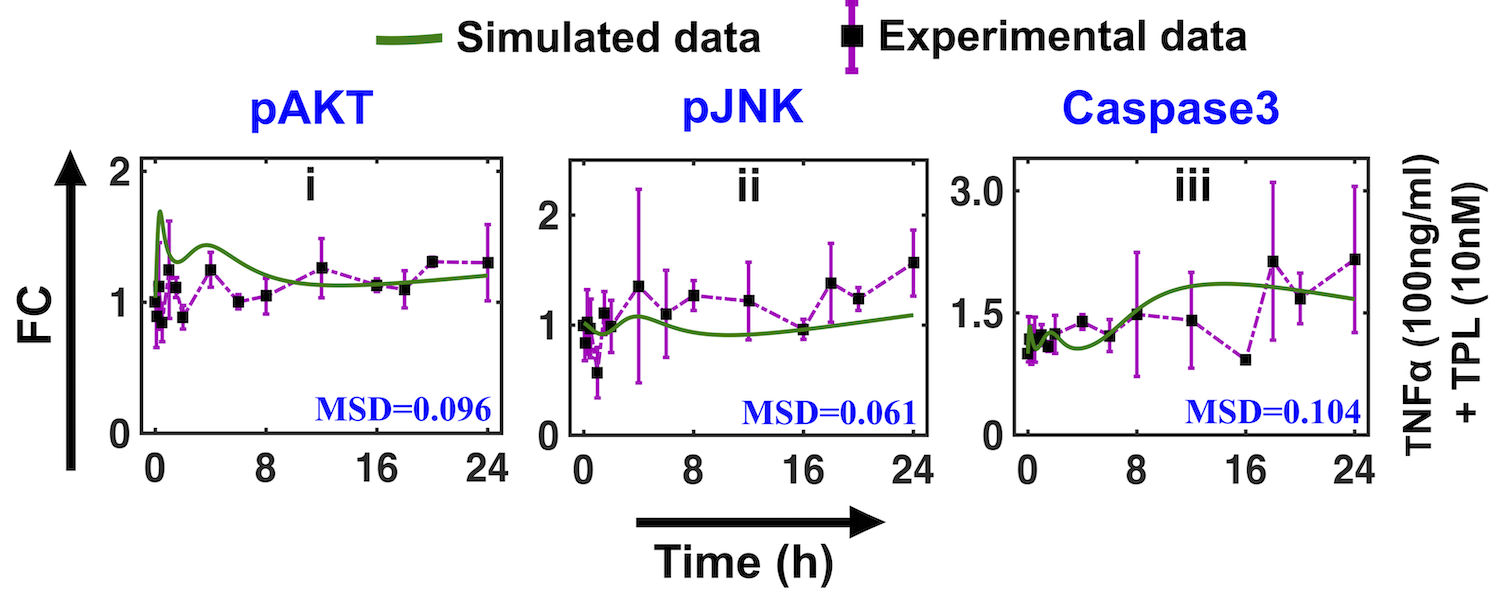

Supplement: S8 Fig — The green lines represent the simulated trajectories and the black dots with corresponding error bars (n = 3) indicate the experimental measurements. (TIFF) [file pcbi.1010626.s015.tiff]

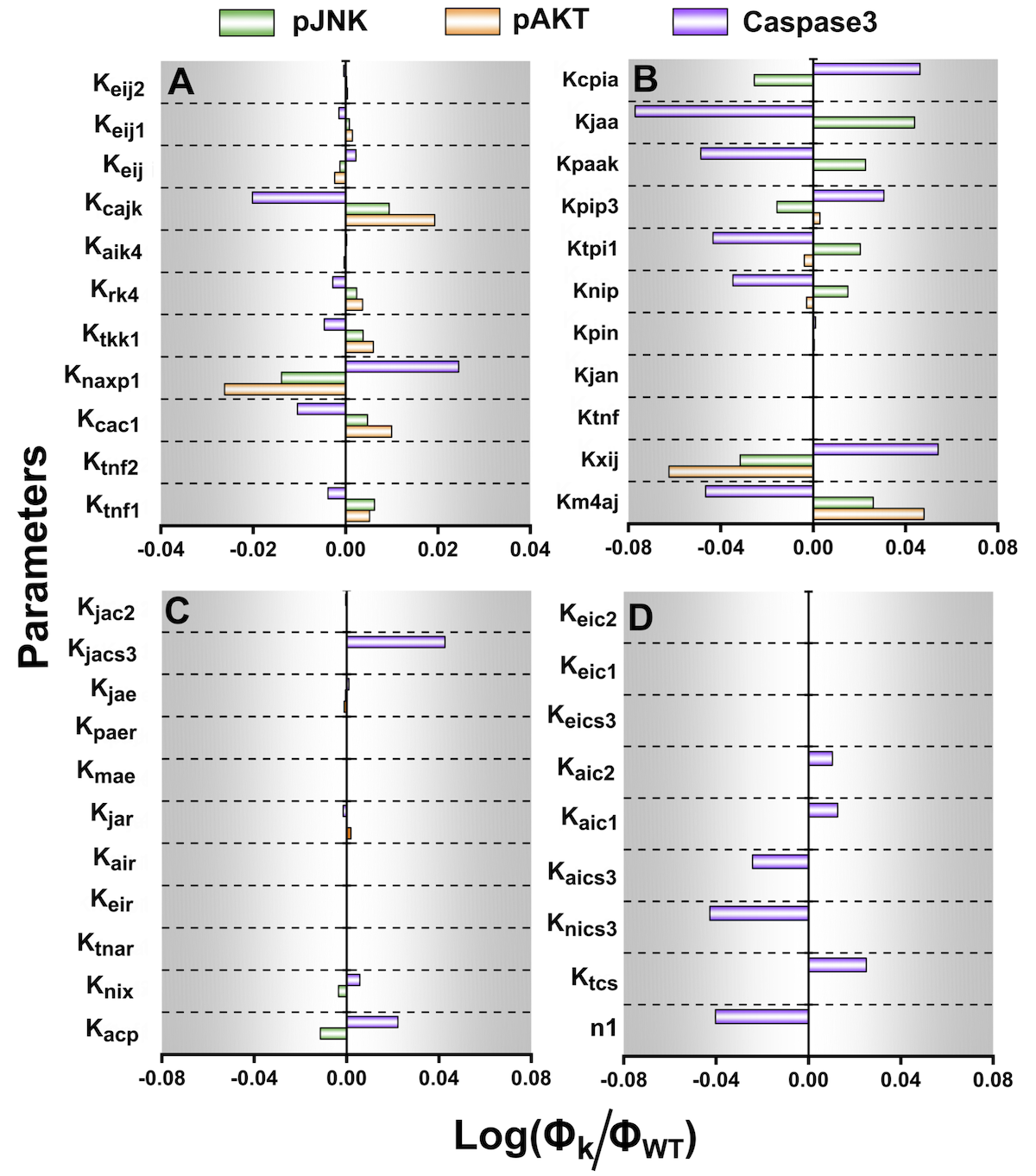

Supplement: S9 Fig — Φk and ΦWT, respectively captures the area under the transient for the case of deviation in a certain cross-talk parameter and that for the case of the best fit parameter. Note that a deviation of 20% (as specified in the third column of Table I in S3 Text) from the best fit values in Table I in S3 Text was considered for this analysis. (TIFF) [file pcbi.1010626.s016.tiff]

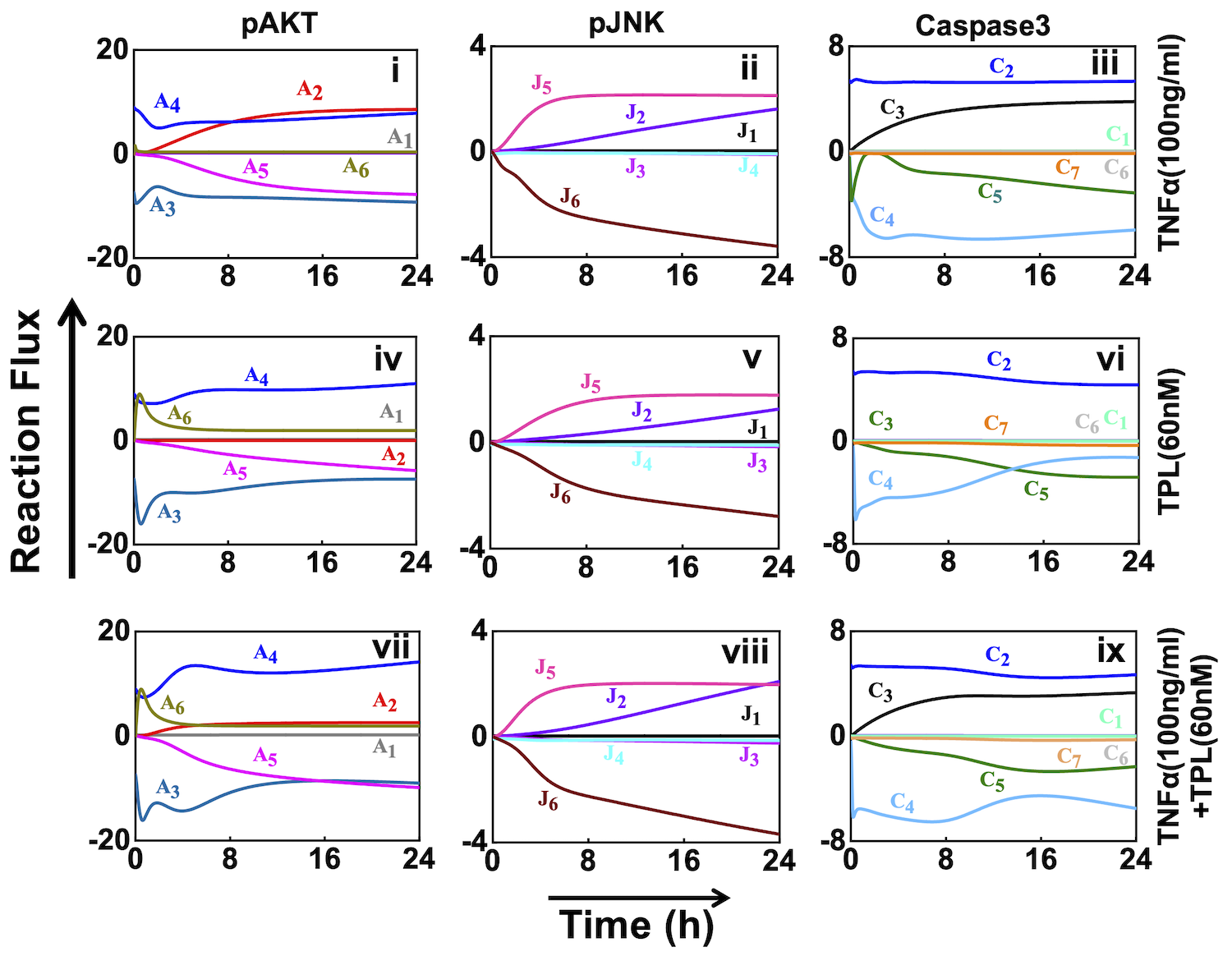

Supplement: S10 Fig — Rows correspond to different stimulation conditions. Expression for these fluxes (Ai, Ji and Ci, for all i) are provided in S4 Table. (TIFF) [file pcbi.1010626.s017.tiff]

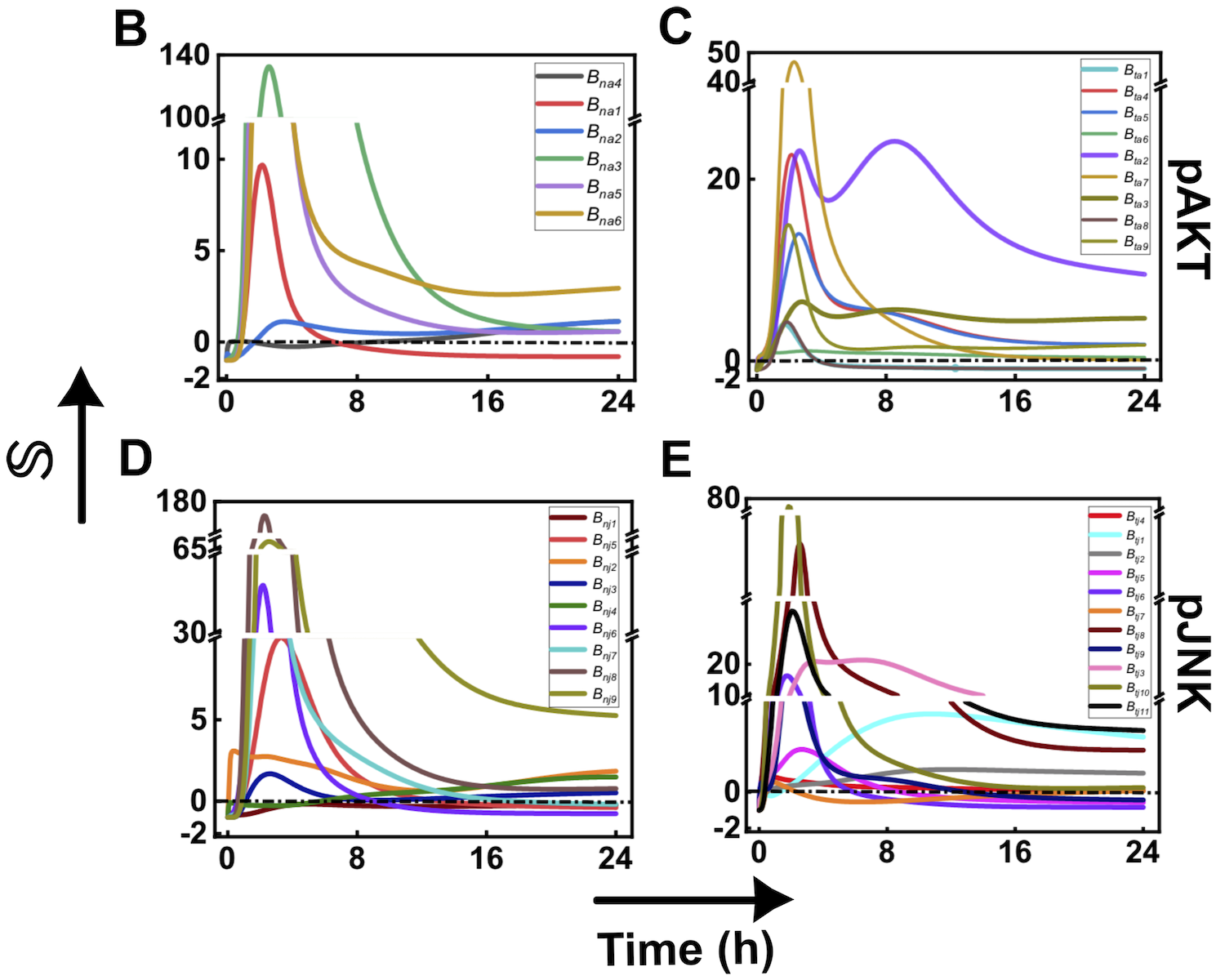

Supplement: S11 Fig — Time-dependent synergism due to all branches from (A) NFκB to pAKT, (B) TNFR1 to pAKT, (C) NFκB to pJNK, and (D) TNFR1 to pJNK as listed in S5 Table. (TIFF) [file pcbi.1010626.s018.tiff]

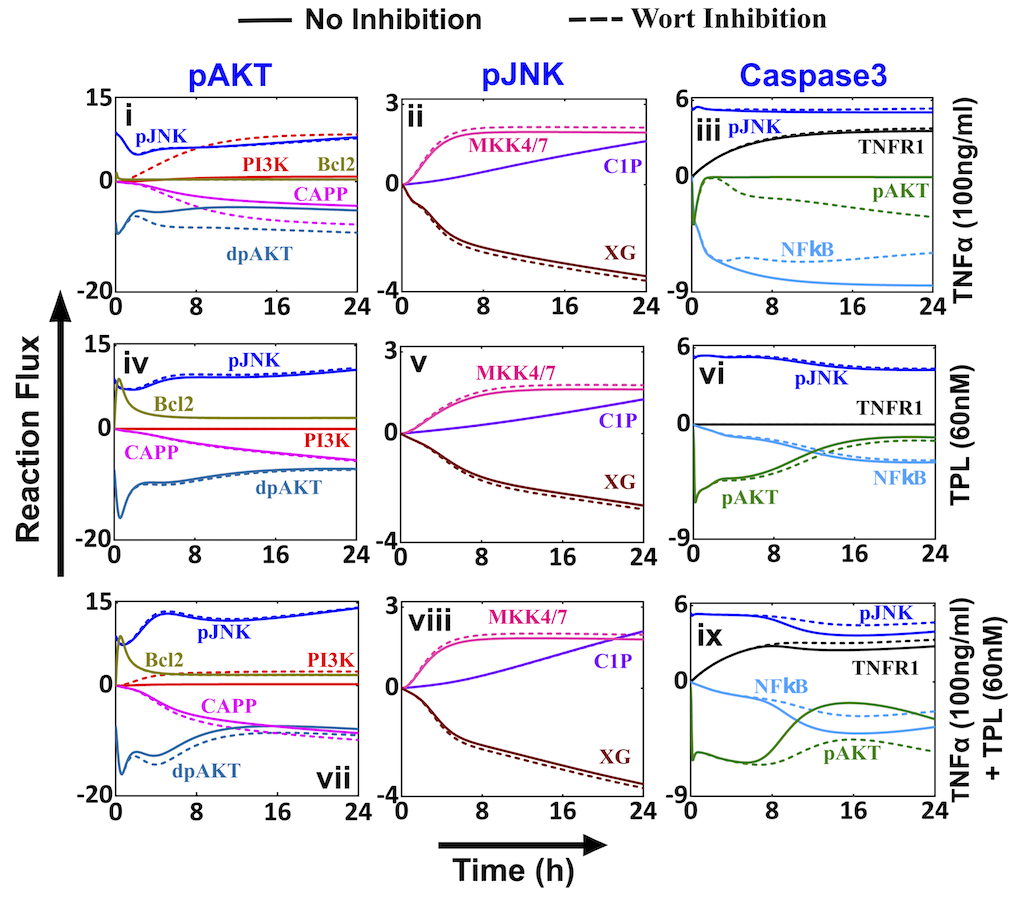

Supplement: S12 Fig — Flux analysis of different nodes on controlling of pAKT, pJNK and Caspase3 under different experimental conditions in the presence of Wort inhibitor. The dotted line represents when simulation has been done at 0 nM (no inhibitor) of Wort and the solid line depicts the trajectories for 1000 nM of Wort. The contribution from each specific node in the time profile of marker protein has been dissected separately by considering various terms in the corresponding model equation in S4 Table. Wherever necessary, the inhibitory action related modifications to the relevant rate terms were considered. Inhibitory parameters used are Kiak = 0.001 nM−1, Kkim = 0.02 nM−1, and Kkxg = 0.00005 nM−1. (TIFF) [file pcbi.1010626.s019.tiff]

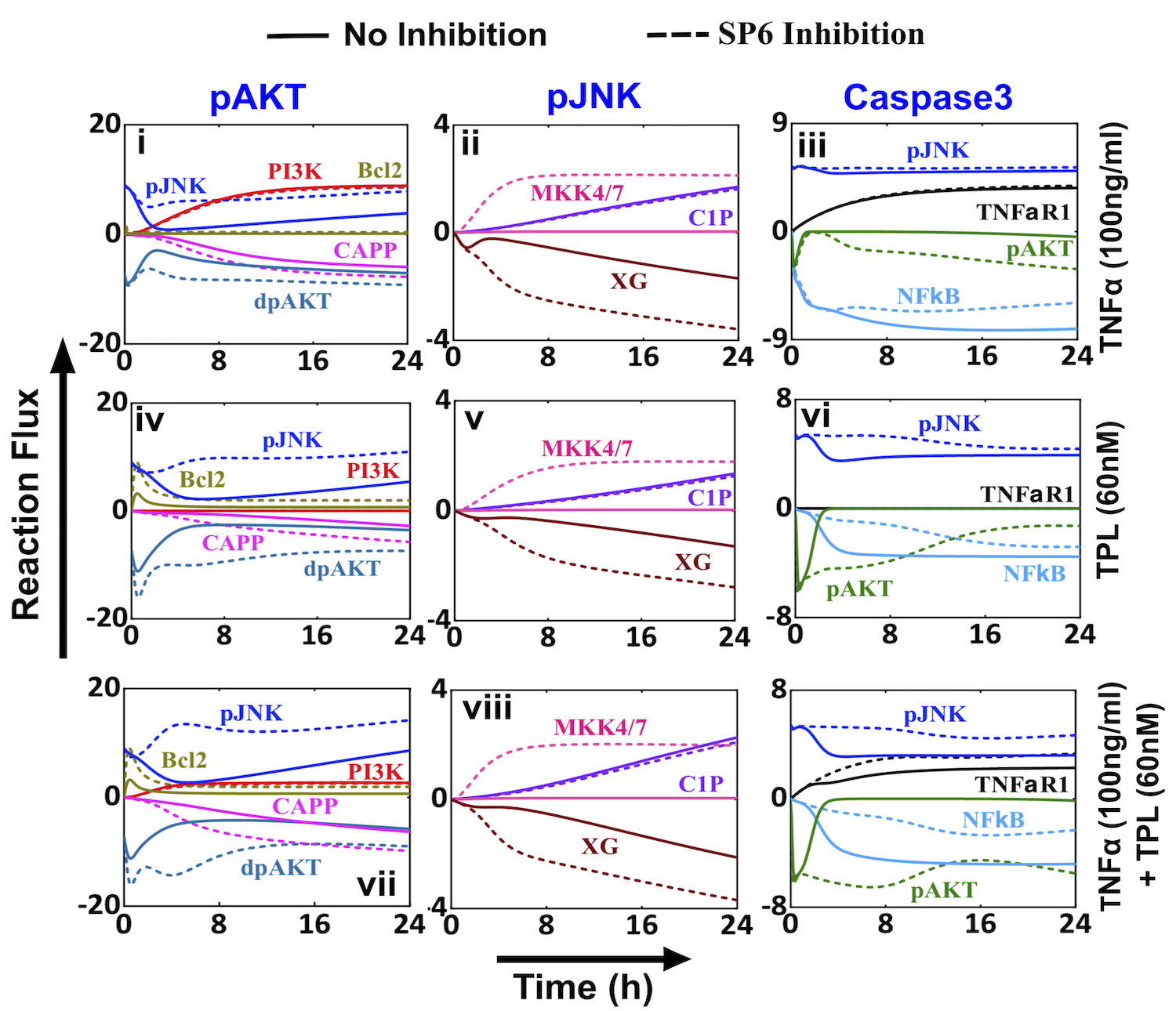

Supplement: S13 Fig — Flux analysis of different nodes on controlling of pAKT, pJNK and Caspase3 under different experimental conditions in the presence of SP6 inhibitor. The dotted line represents when simulation has been done at 0 nM (no inhibitor) SP6 and the solid line depicts the trajectories for 10000 nM of SP6. The contribution from each specific node in the time profile of marker protein has been dissected separately by considering various terms in the corresponding model equation in S4 Table. Wherever necessary, the inhibitory action related modifications to the relevant rate terms were considered. Inhibitory parameters used are Kkib = 0.0002 nM-1 and Kkis = 0.005 nM−1. (TIFF) [file pcbi.1010626.s020.tiff]
