# Supplementary material for: Modulation of signaling cross-talk between pJNK and pAKT generates optimal apoptotic response
Source: PLoS Comput Biol. 2022 Oct 14;18(10):e1010626. doi: 10.1371/journal.pcbi.1010626 (PMC9604984; doi:10.1371/journal.pcbi.1010626)
Supplement: S1 Table — (PDF) [file pcbi.1010626.s021.pdf]

S1 Table: Ordinary differential equations of the TNFα signaling network and associated algebraic relations

Ordinary differential equations	
$$\frac{dTNFR1_a}{dt} = \left((K_{base}) + (K_{tnf1} \times TNFR1 \times TNF) - (K_{tnf2} \times TNFR1_a)\right)$$	[1]
$$\frac{dC1P_a}{dt} = ((K_{bc1} \times C1P) + (K_{cac1} \times C1P \times CER_a) - (K_{cac2} \times C1P_a))$$	[2]
$$\frac{dXG_a}{dt} = \left((K_{bxg} \times XG) + (K_{naxp1} \times XG \times NFkB_a) - \left((K_{dxg} \times XG_a)\right)\right)$$	[3]
$$\frac{dMKK_a}{dt} = ((K_{bkk1} \times MKK) + (K_{tkk1} \times MKK \times TNFR1_a) - (K_{dkk1} \times MKK_a) + (K_{rak4} \times MKK \times ROS_a) - (K_{aik4} \times MKK_a \times pAKT))$$	[4]
$$\frac{dpJNK}{dt} = \left((K_{bjnk} \times JNK) + (K_{cajk} \times JNK \times C1P_a) - \left(\frac{K_{eij} \times pERK \times pJNK}{K_{eij1} + K_{eij2} \times pERK}\right) - (K_{djnk} \times pJNK) + (K_{m4aj} \times JNK \times MKK_a) - (K_{xij} \times XG_a \times pJNK)\right)$$	[5]
$$\frac{dNFkB_a}{dt} = \left(\left[\frac{(K_{bnf} \times NFkB)}{(1 + K_{inh} \times TPL)}\right] + \left[\frac{(K_{tnf} \times NFkB \times TNFR1_a)}{(1 + K_{inh} \times TPL)}\right] - (K_{pin} \times PTEN_a \times NFkB_a) + (K_{jan} \times pJNK \times NFkB) - (K_{dnf} \times NFkB_a)\right)$$	[6]
$$\frac{dPI3K_a}{dt} = \left((K_{bp3k} \times PI3K) + (K_{tpi1} \times PI3K \times TNFR1_a) - (K_{pip3} \times PI3K_a \times PTEN_a) - (K_{bdp3k} \times PI3K_a)\right)$$	[7]
$$\frac{dPTEN_a}{dt} = \left((K_{bpt} \times PTEN) - (K_{nip} \times NFkB_a \times PTEN_a) - (K_{dpt} \times PTEN_a)\right)$$	[8]
$$\frac{dpAKT}{dt} = \left((K_{bak} \times AKT) + (K_{paak} \times PI3K_a \times AKT) - (K_{bdak} \times pAKT) + (K_{jaa} \times pJNK \times AKT) - (K_{cpia} \times CAPP_a \times pAKT) + (K_{xaa} \times Bcl2_a \times AKT)\right)$$	[9]

$\frac{dCER_a}{dt} = \left((K_{bcer} \times CER) + (K_{tcr} \times CER \times TNFR1_a) - (K_{dcer} \times CER_a) - (K_{picr} \times PI3Ka \times CER_a)\right)$	[10]
$\frac{dCAPP_a}{dt} = \left((K_{bcpp} \times CAPP) + (K_{acp} \times CAPP \times CER_a) - (K_{dcpp} \times CAPP_a)\right)$	[11]
$\frac{dBcl2_a}{dt} = \left((K_{bx} \times Bcl2) - (K_{nix} \times Bcl2_a \times NFkB_a) - (K_{dx} \times Bcl2_a)\right)$	[12]
$\frac{dROS_a}{dt} = \left((K_{bros} \times ROS) + (K_{jar} \times ROS \times pJNK) - (K_{dros} \times ROS_a)\right)$	[13]
$\frac{dRAF_a}{dt} = \left((K_{braf} \times RAF) + (K_{tnar} \times TNFR1_a \times RAF) - (K_{dbraf} \times RAF_a) - (K_{eir} \times RAF_a \times pERK) - (K_{air} \times RAF_a \times pAKT)\right)$	[14]
$\frac{dpERK}{dt} = \left((K_{berk} \times ERK) + (K_{mae} \times ERK \times RAF_a) - (K_{dbrk} \times pERK) + (K_{paer} \times ERK \times PI3K_a) + (K_{jae} \times ERK \times pJNK)\right)$	[15]
$\frac{dCs3_a}{dt} = \left(\left[\frac{(K_{jacs3} \times Cs3 \times pJNK)}{(K_{jac2} + (n1 \times pJNK))}\right] - \left[\frac{(K_{eics3} \times Cs3_a \times pERK)}{(K_{eic1} + (K_{eic2} \times pERK))}\right] - \left[\frac{(K_{aics3} \times Cs3_a \times pAKT^{K_{n2}})}{(K_{aic1} + (K_{aic2} \times pAKT^{K_{n2}}))}\right] + (K_{bcs3} \times Cs3) + (K_{tacs} \times Cs3 \times TNFR1_a) - (K_{nics3} \times Cs3_a \times NFkB_a) - (K_{dbcs3} \times Cs3_a)\right)$	[16]
Algebraic relations	
$TNFR1 = (TNFR1T - TNFR1_a)$	[1]
$CER = (CERT - CER_a)$	[2]
$XG = (XGT - XG_a)$	[3]
$MKK = (MKKT - MKK_a)$	[4]
$JNK = (JNKT - pJNK)$	[5]
$NFkB = (NFkBT - NFkB_a)$	[6]
$PI3K = (PI3KT - PI3K_a)$	[7]
$PTEN = (PTENT - PTEN_a)$	[8]

$AKT = (AKTT - pAKT)$	[9]
$CER = (CERT - CER_a)$	[10]
$CAPP = (CAPPT - CAPP_a)$	[11]
$Bcl2 = (Bcl2T - Bcl2_a)$	[12]
$ROS = (ROST - ROS_a)$	[13]
$RAF = (RAFT - RAF_a)$	[14]
$ERK = (ERKT - pERK)$	[15]
$Cs3 = (Cs3T - Cs3_a)$	[16]
$baAKT = \left(\frac{K_{bak} \times AKTT}{K_{bak} + K_{bdak}}\right)$	[17]
$baJNK = \left(\frac{K_{bjnk} \times JNKT}{K_{bjnk} + K_{bjnk}}\right)$	[18]
$baCs3 = \left(\frac{K_{bcs3} \times Cs3T}{K_{bcs3} + K_{dbcs3}}\right)$	[17]
Observables	
$FC\_AKT = S1 \times \left(\frac{pAKT}{baAKT}\right)$	[1]
$FC\_JNK = S \times \left(\frac{pJNK}{baJNK}\right)$	[2]
$FC\_Cs3 = S2 \times \left(\frac{Cs3_a}{baCs3}\right)$	[3]
