# Supplementary material for: Modulation of signaling cross-talk between pJNK and pAKT generates optimal apoptotic response
Source: PLoS Comput Biol. 2022 Oct 14;18(10):e1010626. doi: 10.1371/journal.pcbi.1010626 (PMC9604984; doi:10.1371/journal.pcbi.1010626)
Supplement: S2 Table — (PDF) [file pcbi.1010626.s022.pdf]

S2 Table: Description of the entities in the TNFα signaling network model and its state

Symbol	Description	
TNF	TNF-α ligand	[1]
$TNFR1$	TNF-α receptor-1	[2]
$TNFR1_a$	Active complex formed by binding of TNF-α ligand with the TNF-α receptor-1	[3]
$C1P$	Inactive form of C1P protein	[4]
$C1P_a$	Phosphorylated form of C1P protein	[5]
XG	Inactive form of XIAP and Gadd45B protein	[6]
XG_a	Active form of XIAP and Gadd45B protein	[7]
MKK	Inactive form of MKK4 and MKK7 protein	[8]
MKK_a	Phosphorylated form of MKK4 and MKK7 protein	[9]
JNK	Inactive form of JNK protein	[10]
$pJNK$	Phosphorylated form of JNK protein	[11]
$NF\kappa B$	Inactive form of NFκB protein	[12]
$NF\kappa B_a$	Active form of NFκB protein	[13]
TPL	Triptolide, inhibitor of NFκB protein	[14]
$PTEN$	Inactive form of PTEN protein	[15]
$PTEN_a$	Active form of PTEN protein	[16]
$PI3K$	Inactive form of PI3K kinase	[17]
$PI3K_a$	Active form of PI3K kinase	[18]
AKT	Inactive form of AKT protein	[19]
$pAKT$	Phosphorylated form of AKT protein	[20]
CER	Inactive form of CERAMIDE protein	[21]
CER_a	Active form of CERAMIDE protein	[22]
$CAPP$	Inactive form of CAPP protein	[23]
$CAPP_a$	Active form of CAPP protein	[24]
$Bcl2$	Inactive form of Bcl2 protein	[25]
$Bcl2_a$	Active form of Bcl2 protein	[26]
RAF	Inactive form of RAF complex	[27]
RAF_a	Phosphorylated form of RAF complex	[28]

ROS	Inactive form of ROS protein	[29]
ROS_a	Active form of ROS protein	[30]
ERK	Inactive form of ERK1/2 protein	[31]
$pERK$	Phosphorylated form of ERK1/2 protein	[32]
$Cs3$	Inactive form of cleaved Caspase-3 protein	[33]
$Cs3_a$	Active form of cleaved Caspase-3 protein	[34]
