# Supplementary material for: Modulation of signaling cross-talk between pJNK and pAKT generates optimal apoptotic response
Source: PLoS Comput Biol. 2022 Oct 14;18(10):e1010626. doi: 10.1371/journal.pcbi.1010626 (PMC9604984; doi:10.1371/journal.pcbi.1010626)
Supplement: S3 Table — (PDF) [file pcbi.1010626.s023.pdf]

S3 Table: Definition of the kinetic parameters involved in the model

Symbol	Descriptions of the Parameter	Unit	
K_{base}	Basal synthesis rate of the TNFα receptor-1	h^{-1}	[1]
K_{tnf1}	Complex ($TNFR1_a$) formation rate or Binding rate of TNFα ligand with TNFα receptor-1	$nM^{-1}h^{-1}$	[2]
K_{tnf2}	Dissociation rate of the active Complex ($TNFR1_a$)	h^{-1}	[3]
K_{bc1}	Basal phosphorylation rate of the C1P protein	h^{-1}	[4]
K_{cac1}	CER_a mediated phosphorylation rate of C1P protein	$nM^{-1}h^{-1}$	[5]
K_{cac2}	Dephosphorylation rate of C1P protein	h^{-1}	[6]
K_{bxg}	Basal activation rate of the XG protein	h^{-1}	[7]
K_{naxp1}	$NF\kappa B_a$ mediated transcriptional activation rate of XG protein	$nM^{-1}h^{-1}$	[8]
K_{dxg}	Deactivation rate of XG protein	h^{-1}	[9]
K_{bkk1}	Basal phosphorylation rate of the MKK protein	h^{-1}	[10]
K_{tkk1}	$TNFR1_a$ mediated phosphorylation rate of MKK protein	$nM^{-1}h^{-1}$	[11]
K_{dkk1}	Dephosphorylation rate of MKK protein	h^{-1}	[12]
K_{rk4}	ROS_a mediated phosphorylation rate of MKK protein	$nM^{-1}h^{-1}$	[13]
K_{aik4}	pAKT mediated dephosphorylation rate of MKK protein	$nM^{-1}h^{-1}$	[14]
K_{bjnk}	Basal phosphorylation rate of the JNK protein	h^{-1}	[15]
K_{cajk}	$C1P_a$ mediated phosphorylation of JNK protein	$nM^{-1}h^{-1}$	[16]
K_{eij}	pERK mediated deactivation rate of JNK protein	$nM^{-1}h^{-1}$	[17]
K_{eij1}	Constant controlling the pERK thresholding effect on pJNK inhibition	-	[18]
K_{eij2}	Constant capturing the saturation effect of pERK on pJNK inhibition	nM^{-1}	[19]
K_{djnk}	Dephosphorylation rate of JNK protein	h^{-1}	[20]

K_{m4aj}	MKK$_a$ mediated phosphorylation of JNK protein	$nM^{-1}h^{-1}$	[21]
K_{xij}	XG$_a$ mediated deactivation rate of JNK protein	$nM^{-1}h^{-1}$	[22]
K_{bnf}	Basal activation rate of the NFκB protein	h^{-1}	[23]
K_{inh}	Coefficient controlling the Tpl mediated NFκB inhibition	nM^{-1}	[24]
K_{bnf}	TNFR1$_a$ mediated activation rate of NFκB protein	$nM^{-1}h^{-1}$	[25]
K_{jan}	pJNK mediated activation rate of NFκB protein	$nM^{-1}h^{-1}$	[26]
K_{pin}	PTEN$_a$ mediated deactivation rate of NFκB protein	$nM^{-1}h^{-1}$	[27]
K_{dnf}	Deactivation rate of NFκB protein	h^{-1}	[28]
K_{bpt}	Basal phosphorylation rate of the PTEN protein	h^{-1}	[29]
K_{nip}	NFκB$_a$ mediated inhibition rate of PTEN protein	$nM^{-1}h^{-1}$	[30]
K_{dpt}	Deactivation rate of PTEN protein	h^{-1}	[31]
K_{bp3k}	Basal phosphorylation rate of PI3K protein	h^{-1}	[32]
K_{tpi1}	TNFR1$_a$ mediated activation rate of PI3Ka protein	$nM^{-1}h^{-1}$	[33]
K_{pip3}	PTEN$_a$ mediated dephosphorylation rate of PI3K protein	$nM^{-1}h^{-1}$	[34]
K_{bdp3k}	Dephosphorylation rate of PI3K protein	h^{-1}	[35]
K_{bak}	Basal phosphorylation rate of AKT protein	h^{-1}	[36]
K_{paak}	PI3K$_a$ mediated phosphorylation rate of AKT protein	$nM^{-1}h^{-1}$	[37]
K_{jaa}	pJNK mediated activation rate of AKT protein	$nM^{-1}h^{-1}$	[38]
K_{cpia}	CAPP$_a$ mediated dephosphorylation rate of AKT protein	$nM^{-1}h^{-1}$	[39]
K_{xaa}	Bcl2$_a$ mediated phosphorylation rate of AKT protein	$nM^{-1}h^{-1}$	[40]
K_{bdak}	Dephosphorylation rate of AKT protein	h^{-1}	[41]
K_{bcer}	Basal phosphorylation rate of CERAMIDE	h^{-1}	[42]

	protein		
K_{tcr}	TNFR1ₐ mediated catalysis rate of CERAMIDE protein	$nM^{-1}h^{-1}$	[43]
K_{picr}	$PI3K_a$ mediated inhibition rate of CERAMIDE protein	$nM^{-1}h^{-1}$	[44]
K_{dcer}	Deactivation rate of CERAMIDE protein	h^{-1}	[45]
K_{bx}	Basal activation rate of Bcl2 protein	h^{-1}	[46]
K_{dx}	Deactivation rate of Bcl2 protein	h^{-1}	[47]
K_{nix}	$NF\kappa B_a$ mediated inhibition rate of Bcl2 protein transcription	$nM^{-1}h^{-1}$	[48]
K_{braf}	Basal phosphorylation rate of RAF protein	h^{-1}	[49]
K_{tnar}	$TNFR1_a$ mediate phosphorylation rate of RAF protein	$nM^{-1}h^{-1}$	[50]
K_{eir}	pERK mediated phosphorylation rate of RAF protein leading to deactivation of RAF	$nM^{-1}h^{-1}$	[51]
K_{air}	pAKT mediated phosphorylation rate of RAF protein leading to deactivation of RAF	$nM^{-1}h^{-1}$	[52]
K_{dbraf}	Deactivation rate of RAF protein	h^{-1}	[53]
K_{bros}	Basal activation rate of ROS protein	h^{-1}	[54]
K_{jar}	pJNK mediated activation rate of ROS protein	$nM^{-1}h^{-1}$	[55]
K_{dros}	Deactivation rate of ROS protein	h^{-1}	[56]
K_{berk}	Basal activation rate of ERK protein	h^{-1}	[57]
K_{mae}	RAF_a mediated activation rate of ERK protein	$nM^{-1}h^{-1}$	[58]
K_{paer}	$PI3K_a$ mediated activation rate of ERK protein	$nM^{-1}h^{-1}$	[59]
K_{jae}	pJNK mediated activation rate of ERK protein	$nM^{-1}h^{-1}$	[60]
K_{dbrk}	Deactivation rate of ERK protein	h^{-1}	[61]
K_{bcs3}	Basal activation rate of Cs3 protein	h^{-1}	[62]
K_{jacs3}	pJNK mediate activation rate of Cs3 protein	h^{-1}	[63]
K_{jac2}	Constant controlling the pJNK thresholding effect on Cs3 activation	-	[64]

$n1$	Constant capturing the saturation effect of pJNK on Cs3 activation	nM^{-1}	[65]
K_{tcs}	$TNFR1_a$ mediate activation rate of Cs3 protein	$nM^{-1}h^{-1}$	[66]
K_{nics3}	$NF\kappa B_a$ mediate deactivation rate of Cs3 protein	nM^{-1} h^{-1}	[67]
K_{aics3}	pAKT mediate deactivation rate of Cs3 protein	$nM^{-K_{n2}}h^{-1}$	[68]
K_{aic1}	Constant controlling the pAKT thresholding effect on Cs3a inhibition	-	[69]
K_{aic2}	Constant capturing the saturation effect of pAKT on Cs3a inhibition	nM^{-1}	[70]
K_{n2}	Phenomenological exponent to pAKT inhibiting Cs3a	-	[71]
K_{eics3}	pERK mediate deactivation rate of Cs3 protein	$nM^{-1}h^{-1}$	[72]
K_{eic1}	Constant controlling the pERK thresholding effect on Cs3a inhibition	-	[73]
K_{eic2}	Constant capturing the saturation effect of pERK on Cs3a inhibition	nM^{-1}	[74]
K_{dbcs3}	Deactivation rate of Cs3 protein	h^{-1}	[75]
K_{bcpp}	Basal activation rate of CAPP protein	h^{-1}	[76]
K_{acp}	CER_a mediated activation of CAPP protein	$nM^{-1}h^{-1}$	[77]
K_{dcpp}	Deactivation rate of CAPP protein	h^{-1}	[78]
S	Scaling factor that scaled the basal level of pJNK protein	-	[79]
$S1$	Scaling factor that scaled the basal level of pAKT protein	-	[80]
$S2$	Scaling factor that scaled the basal level of $Cs3_a$ protein	-	[81]
