# Supplementary material for: Modulation of signaling cross-talk between pJNK and pAKT generates optimal apoptotic response
Source: PLoS Comput Biol. 2022 Oct 14;18(10):e1010626. doi: 10.1371/journal.pcbi.1010626 (PMC9604984; doi:10.1371/journal.pcbi.1010626)
Supplement: S4 Table — (PDF) [file pcbi.1010626.s024.pdf]

S4 Table: Reaction fluxes contributing to pJNK, NFκB, pAKT and Caspase3 dynamics

$J_i \forall i = 1,6$ indicates fluxes corresponding to different rates in Eq 5 in S1 Table $$\frac{dpJNK}{dt} = (J_1 + J_2 + J_3 + J_4 + J_5 + J_6)$$	
$J_1 = (K_{bjnk} \times JNK)$	[1]
$J_2 = (K_{cajk} \times JNK \times C1P_a)$	[2]
$J_3 = -\left(\frac{K_{eij} \times pERK \times pJNK}{K_{eij1} + K_{eij2} \times pERK}\right)$	[3]
$J_4 = -(K_{djnk} \times pJNK)$	[4]
$J_5 = (K_{m4aj} \times JNK \times MKK_a)$	[5]
$J_6 = -(K_{xij} \times XG_a \times pJNK)$	[6]
$N_i \forall i = 1,5$ indicates fluxes corresponding to different rates in Eq 6 in S1 Table $$\frac{dNF\kappa B}{dt} = (N_1 + N_2 + N_3 + N_4 + N_5)$$	
$N_1 = \left[\frac{(K_{bnf} \times NFkB)}{(1 + K_{inh} \times TPL)}\right]$	[1]
$N_2 = \left[\frac{(K_{tnf} \times NFkB \times TNFR1_a)}{(1 + K_{inh} \times TPL)}\right]$	[2]
$N_3 = -(K_{pin} \times PTEN_a \times NFkB_a)$	[3]
$N_4 = (K_{jan} \times pJNK \times NFkB)$	[4]
$N_5 = -(K_{dnf} \times NFkB_a)$	[5]
$A_i \forall i = 1,6$ indicates fluxes corresponding to different rates in Eq 9 in S1 Table $$\frac{dpAKT}{dt} = (A_1 + A_2 + A_3 + A_4 + A_5 + A_6)$$	
$A_1 = (K_{bak} \times AKT)$	[1]
$A_2 = (K_{paak} \times PI3K_a \times AKT)$	[2]
$A_3 = -(K_{bdak} \times pAKT)$	[3]
$A_4 = (K_{jaa} \times pJNK \times AKT)$	[4]
$A_5 = -(K_{cpia} \times CAPP_a \times pAKT)$	[5]
$A_6 = (K_{xaa} \times Bcl2 \times AKT)$	[6]
$C_i \forall i = 1,7$ indicates fluxes corresponding to different rates in Eq. 16 in S1 Table $$\frac{dCs3_a}{dt} = (C_1 + C_2 + C_3 + C_4 + C_5 + C_6 + C_7)$$	

$C_1 = (K_{bcs3} \times Cs3)$	[1]
$C_2 = \left(\frac{K_{jacs3} \times Cs3 \times pJNK}{K_{jac2} + n1 \times pJNK}\right)$	[2]
$C_3 = (K_{tcs} \times Cs3 \times TNFR1_a)$	[3]
$C_4 = -(K_{nics3} \times Cs3_a \times NF\kappa B_a)$	[4]
$C_5 = -\left(\frac{K_{aics3} \times Cs3 \times pAKT^{K_{n2}}}{K_{aic1} + K_{aic2} \times pAKT^{K_{n2}}}\right)$	[5]
$C_6 = -\left(\frac{K_{eics3} \times Cs3_a \times pERK}{K_{eic1} + K_{eic2} \times pERK}\right)$	[6]
$C_7 = -(K_{dbcs3} \times Cs3_a)$	[7]
