# Supplementary material for: Modulation of signaling cross-talk between pJNK and pAKT generates optimal apoptotic response
Source: PLoS Comput Biol. 2022 Oct 14;18(10):e1010626. doi: 10.1371/journal.pcbi.1010626 (PMC9604984; doi:10.1371/journal.pcbi.1010626)
Supplement: S5 Table — (PDF) [file pcbi.1010626.s025.pdf]

S5 Table: Branches originating from NFκB or TNFR1 and ending in the signaling entities pAKT or pJNK.

ID	Branch
B_{na1}	NFκB ⊣ PTEN ⊣ PI3K → AKT
B_{na2}	NFκB → XG ⊣ JNK → AKT
B_{na3}	NFκB ⊣ PTEN ⊣ PI3K → ERK1/2 ⊣ JNK → AKT
B_{na4}	NFκB ⊣ B-cl2 → AKT
B_{na5}	NFκB ⊣ PTEN ⊣ PI3K ⊣ Ceramide → CAPP ⊣ AKT
B_{na6}	NFκB ⊣ PTEN ⊣ PI3K ⊣ Ceramide → C1P → JNK → AKT
B_{ta1}	TNFR1a → PI3K → AKT
B_{ta2}	TNFR1a → RAF → ERK1/2 ⊣ JNK → AKT
B_{ta3}	TNFR1a → Ceramide → C1P → JNK → AKT
B_{ta4}	TNFR1a → MKK4/7 → JNK → AKT
B_{ta5}	TNFR1a → Ceramide → CAPP ⊣ AKT
B_{ta6}	TNFR1a → MKK4/7 → JNK → ROS → MKK4/7
B_{ta7}	TNFR1a → PI3K → ERK1/2 ⊣ JNK → AKT
B_{ta8}	TNFR1a → PI3K ⊣ Ceramide → CAPP ⊣ AKT
B_{ta9}	TNFR1a → PI3K ⊣ Ceramide → C1P → JNK → AKT
B_{nj1}	NFκB → XG ⊣ JNK
B_{nj2}	NFκB ⊣ Bcl-2 → AKT ⊣ MKK4/7 → JNK
B_{nj3}	NFκB ⊣ PTEN ⊣ PI3K → Ceramide → C1P → JNK
B_{nj4}	NFκB ⊣ Bcl-2 → AKT → RAF → ERK1/2 ⊣ JNK
B_{nj5}	NFκB ⊣ PTEN ⊣ PI3K → ERK1/2 ⊣ JNK
B_{nj6}	NFκB ⊣ PTEN ⊣ PI3K → AKT ⊣ MKK4/7 → JNK
B_{nj7}	NFκB ⊣ PTEN ⊣ PI3K → AKT → RAF → ERK1/2 ⊣ JNK
B_{nj8}	NFκB ⊣ PTEN ⊣ PI3K ⊣ Ceramide → CAPP ⊣ AKT ⊣ MKK4/7 → JNK
B_{nj9}	NFκB ⊣ PTEN ⊣ PI3K ⊣ Ceramide → CAPP ⊣ AKT ⊣ RAF → ERK1/2 ⊣ JNK
B_{tj1}	TNFR1a → RAF → ERK1/2 ⊣ JNK
B_{tj2}	TNFR1a → Ceramide → C1P → JNK
B_{tj3}	TNFR1a → Ceramide → CAPP → AKT ⊣ RAF → ERK1/2 ⊣ JNK
B_{tj4}	TNFR1a → MKK4/7 → JNK
B_{tj5}	TNFR1a → PI3K → ERK1/2 ⊣ JNK
B_{tj6}	TNFR1a → PI3K → AKT ⊣ MKK4/7 → JNK
B_{tj7}	TNFR1a → PI3K ⊣ Ceramide → C1P → JNK
B_{tj8}	TNFR1a → Ceramide → CAPP ⊣ AKT ⊣ MKK4/7 → JNK
B_{tj9}	TNFR1a → PI3K → AKT → RAF → ERK1/2 ⊣ JNK
B_{tj10}	TNFR1a → PI3K ⊣ Ceramide → CAPP ⊣ AKT ⊣ MKK4/7 → JNK
B_{tj11}	TNFR1a → PI3K ⊣ Ceramide → CAPP ⊣ AKT ⊣ RAF → ERK1/2 ⊣ JNK
