# Supplementary material for: Modulation of signaling cross-talk between pJNK and pAKT generates optimal apoptotic response
Source: PLoS Comput Biol. 2022 Oct 14;18(10):e1010626. doi: 10.1371/journal.pcbi.1010626 (PMC9604984; doi:10.1371/journal.pcbi.1010626)
Supplement: S6 Table — (PDF) [file pcbi.1010626.s026.pdf]

<u>S6 Table</u>. Coefficients of the fourth order polynomial (Eq S7.1, S7 Text) under different stimulation conditions.

Stimulation condition	Coefficients in Eq. (S7.1)					R^2 (%)	χ^2
	A0	A1	A2	A3	A4		
**Set 1**							
TNFα	−11.78	291.10	−1014.19	1371.01	−610.14	99.43	1.865
TPL	−49.18	783.79	−2078.08	2332.93	−914.35	99.99	0.002
TNFα+TPL	−21.72	697.59	−2218.51	2831.02	−1208.87	99.98	0.097
**Set 2**							
TNFα	−12.41	297.91	−1036.15	1397.82	−621.17	99.39	1.995
TPL	−52.28	823.35	−2218.04	2519.61	−997.54	99.99	2.24×10^{-4}
TNFα+TPL	−21.52	693.89	−2200.04	2797.99	−1190.82	99.99	0.091
**Set 3**							
TNFα	−11.81	291.41	−1015.06	1371.97	−610.52	99.43	1.867
TPL	−50.24	791.86	−2098.19	2356.04	−924.36	99.99	0.010
TNFα+TPL	−20.99	691.19	−2190.43	2779.57	−1179.84	99.99	0.083
