# Supplementary material for: Modulation of signaling cross-talk between pJNK and pAKT generates optimal apoptotic response
Source: PLoS Comput Biol. 2022 Oct 14;18(10):e1010626. doi: 10.1371/journal.pcbi.1010626 (PMC9604984; doi:10.1371/journal.pcbi.1010626)
Supplement: Attachment — Submitted filename: ResponseToReviewersComments.pdf [file pcbi.1010626.s027.pdf]

Response to Editor's and Reviewers' comments

E1: *Dear Dr. Viswanathan,*

Thank you very much for submitting your manuscript "Modulation of signaling cross-talk between pJNK and pAKT generates optimal apoptotic response" for consideration at PLOS Computational Biology. As with all papers reviewed by the journal, your manuscript was reviewed by members of the editorial board and by several independent reviewers. In light of the reviews (below this email), we would like to invite the resubmission of a significantly-revised version that takes into account the reviewers' comments. We cannot make any decision about publication until we have seen the revised manuscript and your response to the reviewers' comments. Your revised manuscript is also likely to be sent to reviewers for further evaluation. When you are ready to resubmit, please upload the following: [1] A letter containing a detailed list of your responses to the review comments and a description of the changes you have made in the manuscript. Please note while forming your response, if your article is accepted, you may have the opportunity to make the peer review history publicly available. The record will include editor decision letters (with reviews) and your responses to reviewer comments. If eligible, we will contact you to opt in or out.[2] Two versions of the revised manuscript: one with either highlights or tracked changes denoting where the text has been changed; the other a clean version (uploaded as the manuscript file).Important additional instructions are given below your reviewer comments.Please prepare and submit your revised manuscript within 60 days. If you anticipate any delay, please let us know the expected resubmission date by replying to this email. Please note that revised manuscripts received after the 60-day due date may require evaluation and peer review similar to newly submitted manuscripts.Thank you again for your submission. We hope that our editorial process has been constructive so far, and we welcome your feedback at any time. Please don't hesitate to contact us if you have any questions or comments.Sincerely,

Jing Chen

Guest EditorPLOS Computational Biology

Douglas Lauffenburger

Deputy Editor

PLOS Computational Biology

Response: We would like to thank the guest editor and the deputy editor for having our manuscript reviewed and also giving us the opportunity to submit a revised version. We sincerely thank all the reviewers for their critical comments which has improved the manuscript significantly. We have now incorporated these suggestions in the relevant sections of the revised manuscript. Please find below a detailed point-wise response along with reference to the changes, if any incorporated in the manuscript.

Reviewer #1:

R1.1: *In this study, Biswas et al. studied signaling cross-talks between pJNK and pAKT pathways on cell apoptosis decision of a U937 cell line using data-driven mathematical modeling and quantitative measurements. The authors have performed extensive studies with various analysis methods to provide mechanistic information on how the two pathways may synergetically affect Caspase3 level and therefore affects cellular apoptosis. The results are in general solid and have the potential to expand our understanding of this important cell fate decision process. However I feel that the presentation of this work needs substantial revision to convey more effectively on the message the authors want the readers to receive. Here are some main comments.*

Response: We sincerely thank the reviewer for the positive comments, critical assessment, and suggestions. We have now addressed all these queries.

R1.2: *Despite all the extensive analyses, at the end I can't find a distilled mechanistic summary that can provide an intuitive and transparent picture to general readers on how the transient dynamic profiles of the two pathways fine-tune the Caspase3 activation.*

Response: We thank the reviewer for this suggestion. We now introduce a figure (Figure 8, main text; see below) detailing the mechanistic summary of how the transient dynamic synergism of the different branches regulating pAKT and pJNK fine-tune the Caspase3 activation and thereby apoptotic response. Further these are discussed in a new paragraph in the revised manuscript "For U937 cells, we identified that the influence in the early phase (< 8 hours) is distinct to that in the late phase (8 to 24 hours) (Fig 8). This was deciphered by tracking the nature of synergism in signal flow via different branches using the branch analysis (Fig. 4). For example, the signal transduction requires NFκB → pAKT branch to dynamically switch from a positive to negative synergism across the two phases (Fig. 8). The low Caspase3 levels at the early phase and the

subsequent rise in the late phase (Fig 2) can be attributed to the early phase negative synergism in the NFκB → pJNK branch being absent in the late phase (Fig 8). Moreover, the positive synergism elicited by the branches originating from TNFR1 involving the RAF-ERK1/2-JNK positive feedback loop influences the dynamics of pAKT in both early and late phases (Fig 8). Combined effect of the dynamically varying synergism in these branches governs the Caspase3 transients via pAKT and pJNK, and therefore the phenotypic apoptotic response (Fig 5B).” in the “Discussion and Conclusion” section in page 25, lines 595-606.

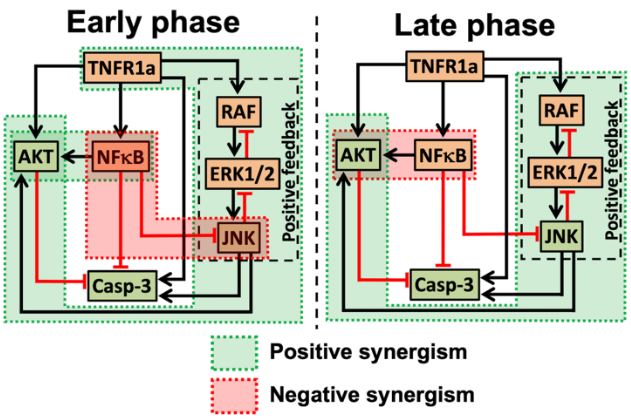

Figure 8: Comparison of time-dependent synergism during the early and late phase signaling cross-talk regulating Caspase3 activation.

R1.3: *1a: On page 6, paragraph: For later time, the apoptosis levels are similar for TPL treatment and TPL + TNFα treatment; but for earlier time (4h, and to a less extent 8h), the combined treatment leads to an apoptosis level more or less the sum of individual TPL and TNFα treatment. I think this observation is the phenomenon that the authors want to address. Am I correct?*

Response: Yes, the primary objective of the manuscript is to unravel the mechanism that could explain this observation highlighted by the reviewer. We have now explicitly stated so in the revised manuscript in the sentence “Overall, in the early phase (<8 hrs), the apoptosis level during the combined treatment is nearly equal to the sum of that achieved for individual TPL and TNFa stimulation. On the contrary, in the later phase (>8 hrs), the apoptosis levels for TPL and TPL+TNFa treatment are nearly equal. However, the exact control mechanism governing this coordination and synergism remains unclear.” in page 7, line 137-142.

R1.4: *1b: As indicated in Fig. 1B, TNFα triggers both death and survival paths (through four parallel pathways), so the final decision is a tug-of-war between the two sub-programs. TPL treatment tips the balance towards the death fate by weakening/blocking the survival program --- but it should be only partially since inhibition on Caspas3 is through either pAKT or NFkB. Is this simplified network sufficient to explain the results?*

Response: We agree with the reviewer that TPL pre-treatment tips the balance towards the cell-death fate. As suggested in **R1.5** (see below), we performed a detailed branch analysis of the extended network in Fig. 2A which clearly demonstrated that the various pathways lumped in Fig. 1B are crucial in regulating the cross-talk signaling that modulates the Caspase3 response leading to apoptosis. This implies that the simplified network in Fig. 1B is insufficient to completely understand the cross-talk regulation dictating the experimental observations. We have highlighted this in the sentence "Moreover, the synergism in eliciting distinct apoptotic responses in the early and later phases cannot be determined using the simple network proposed in Fig. 1B." in page 8, lines 176-177 of the revised manuscript.

R1.5: *1c: How (or can) the temporal dynamics of (i.e., propagation along) each branch explain the crosstalk and resultant apoptosis level?*

Response: We thank the reviewer for raising this important question. We have now performed a detailed branch analysis to distill out the extent of signal transferred between key nodes of the proposed network in Fig. 2A. We introduced the section "Branch analysis reveals the synergistic dynamic cross-talk signaling regulating Caspase3 transients" in page 14 which includes a new figure (Fig. 4) detailing the synergistic signal transfer dynamics. While that due to most influential branches, listed in a new table (Table 1), are presented in Fig. 4, those due to rest of the branches (S5 Table) are in S11 Fig.

R1.6: *1d: Do the temporal profiles shown in Fig. 2B provide clue on the "synergy" (? The terms means that the response from a combined treatment is larger than the sum of responses of individual treatments)?*

Response: We thank the reviewer for raising this interesting point. Fig. 4 and S11 Fig containing the branch analysis introduced in the revised manuscript shows the extent of synergism achieved via different branches due to the combined treatment. The analysis further showcased an important

feature of dynamically varying synergism indicating that the signal is routed through different branches in a time-dependent manner. This is highlighted in the new section entitled as "Branch analysis reveals the synergistic dynamic cross-talk signaling regulating Caspase3 transients" in page 14 in the revised manuscript.

R1.7: *1e: What molecular species serve as the converging nodes?*

Response: The branch analysis revealed that NFκB and PI3K are serving as the influencing converging nodes that primarily control the synergistic behavior. Moreover, the feedback module RAF-ERK-JNK serves as a converging motif regulating the extent of cross-talk signaling. We have highlighted this while discussing the branch analysis in the following sentences in the revised manuscript in the sentences "Branch B_{na1} originating from $NF\kappa B$ offers positive synergism in the early phase and switches to negative synergism beyond 8 hrs (Fig. 4A). In particular, this positive synergism corresponds to the early rise of pAKT transient response for the $TNF\alpha + TPL$ (Fig. 2B-vii), which could be attributed to the effect of TPL via PI3K." in page 15, lines 341-343 and also "The presence of RAF-ERK-JNK positive feedback in branch B_{nj4} could have resulted in the multiple rise and drop of synergism." in page 16, lines 364-366.

R1.8: *1f: While they used the detailed wiring diagram in Fig 2A for model studies, is it really necessary to use such a detailed model? Even so, can they also provide a more coarse-grained network structure just for understanding, so one can have a more transparent picture on how the network structure leads to specific form of the Caspase3 dynamics they observed? Feedback and feed-forward loops, bifurcation analyses, etc., which may help on the understanding? Is the network in Fig. 1B sufficient to summarize the basic structure of the network in Fig. 2B?*

Response: Branch analysis clearly shows that the basic structure in Fig. 1B is insufficient to explain the dynamic signaling cross-talk behavior as feedback loops (RAF-ERK-JNK) and feedforward loop (NFkB-XG-JNK) control them. Branch analysis reveals strong inter-relationship between different distant branches, which will be lost upon coarse-graining. We have discussed these in the paragraph starting "Overall the branch analysis…" in page 16, lines 375-382 of the revised manuscript. To elucidate the key transient inter-dependencies that control the overall cross-talk signaling, as also suggested in comment **R1.2**, we now present a summary figure (Fig. 8)

showcasing a coarse-grained network structure highlighting the important branches regulating Caspase3 dynamics.

R1.9: *Comment 2. For Fig.1Cii-iii, the case of TNFα stimulation only, the authors state that there is an initial increase in the fold change in pJNK and pAKT levels (represented in blue bars). First of all, the trajectories are too packed/cramped in which the increases in fold change are really hard to see (the color bars are tangled together especially in the initial 0- 8h).*

Response: We agree that the trajectories are too packed in the initial phase. Thus, we have provided an inset (in Fig. 1Ci-iii) which shows the zoomed version of the initial phase (0-4h) dynamics. This is also mentioned in the phrase "insets show the zoomed version for the first 4 hours" in Fig 1 caption, page 8, line 165.

R1.10: *2b: Also, with TNFα and TPL treatment (black bars) in Fig.1Ciii, why are the levels of pAKT in TPL only and TPL + TNFα experiments that high compare to TNFα only treatment (since the level of pAKT is not affected by TPL and the level of NFκB)?*

Response: NFκB regulates pAKT via multiple pathways, some positively (e.g. NFκB-PTEN-PI3K-pAKT) and rest negatively (e.g. NFκB-XG-pJNK-pAkt and NFκB-BCL2-pAKT). When cells are treated with TPL causing NFκB inhibition, the NFκB-PTEN-PI3K-pAKT does not contribute to pAKT levels as shown by the flux from PI3K (Fig. 3B-i (red)). However, the basal BCL2 and JNK, whose inhibition due to NFκB is absent, contribute to the increased pAKT levels during TPL treatment (Fig. 3B-I (blue and magenta). While contributions from BCL2 (i.e., via NFκB-BCL2-pAKT) is high only in the initial phase, that from JNK (i.e., via NFκB-XG-JNK-pAKT) is sustained for the entire duration (Fig. 3B-I (blue and magenta)). Therefore, the pAKT levels in the case of TPL are higher compared to that of TNFα treatment. Since the pAKT dynamics is primarily governed by TPL, the TNFα+TPL treatment as well shows higher pAKT levels as compared to only TNFα case. This is discussed in the sentences "Thus, pAKT levels in the case of TPL are higher compared to that of TNFα treatment. Since pAKT dynamics is primarily governed by TPL, the TNFα+TPL treatment as well shows higher pAKT levels as compared to only TNFα case." in page 12, lines 283-285 in the revised manuscript.

R1.11: *Comment 3. In Fig. 5 where the U937 cells were treated with either Wort or SP6, the authors state that the experimentally measured pAKT levels and Caspase3 levels match well with their modeling results. However, for the TPL only and TNFα + TPL cases, the modeling results do not seem to correlate well with that in experimental data. Also, there is a discrepancy between the modeling results and experimental results on pJNK levels, as also stated by the authors themselves, that the "Wort inhibitory model underestimated the pJNK level (Fig. 5Dii)". There might be a typo when the authors refer to this discrepancy, as they said the Wort inhibitory model underestimates the pJNK levels, and use Fig 5Aii, blue as its reference. Do they refer to Fig. 5Dii, blue, since Fig 5A is a schematic representing the Wort inhibitory mechanism. The discrepancy is also present in the SP6 inhibitory model (Fig. 5Dii and Fig. 5Dv). It would be better if the authors can explain more on the discrepancy between the modeling results and the experimental results (e.g. why did over- or underestimation happen).*

Response: We thank the reviewer for bringing these discrepancies to our notice. We have now cited the correct figure number in the text and also thoroughly checked for incorrect citations everywhere. We further revisited the analysis and noted that Wortamannin can also inhibit the activation of XG by NFκB (Manna and Agarwal, 2000 [reference 67 in the revised manuscript]). We repeated the simulations under Wort inhibitory conditions by adding this inhibitory action and found that the predictions improved for pAKT and Caspase3. In particular the transient rise in the Caspase3 under TPL and TNFα+TPL conditions show a similar trend as that of experiments (old Fig. 5 and updated Fig. 6 in the revised manuscript).

Experiments show that transient Caspase3 rise correlates with drop in the pAKT transient under TPL and TNFα+TPL conditions. However, for these two conditions, in the model predictions, the pAKT transients are maintained at a higher steady-level, which is above that for the case of only TNFα treatment. This corroborates why model underestimates the relative rise in the Caspase3 transients for TPL and TNFα+TPL treatment under Wort inhibitory conditions. We further show that pJNK flux maintains a higher pAKT levels at later time points. We have highlighted this causality in the revised manuscript in pages 21-22, lines 503-509.

Moreover, in the case of SP6 inhibitory conditions, the model predicts an early dip in the pAKT transient (caused by complete shutdown of MKK4/7 mediated pJNK activation), which, along with the dip in pJNK level are collectively responsible for the early rise in the caspase3 levels as compared to experimental observations. Note that the model correctly predicts that the

transient dip in pAKT, pJNK and therefore rise in Caspase3 for the TPL and TNFα+TPL cases. The flux analysis reveals that the contributions from pJNK is responsible for the dip and rise in the transients concomitantly. This is discussed in detail in the revised manuscript in page 22, lines 510-517.

The dynamics of pJNK is dictated, among others, by a few nested positive and negative feedback loops involving RAF and pERK. Unfortunately, the model based parameter estimation did not offer a good estimate of the kinetic parameters such as Kmae, Keir, Kair (S9 Fig) which control the flux towards pJNK from these nested feedback loops working via RAF and pERK. Therefore, the dynamic state of the pJNK is poorly represented during the model parameter estimation which explains the observed discrepancy in the model prediction for pJNK under inhibitory conditions. We discuss these points in the revised manuscript in page 21, lines 485-488.

R1.12: *3a: For the inhibitory models along with inhibitory experiments shown in Fig. 5, although Wort and SP6 inhibit both pAKT and pJNK activation through different pathways, their functions are basically the same as the goal is to inhibit pAKT and pJNK activations. Is it necessary to perform two separate modeling and experimental studies considering their blocking targets are the same?*

Response: We thank the reviewer for this suggestion. We note that Wortamannin can also inhibit the activation of XG by NFκB (Manna and Aggarwal, 2000 [reference 67 in the revised manuscript]), which we inadvertently missed in the earlier version. The inhibitory action of Wort on NFκB mediated XG activation could influence the signal flow through network in a qualitative manner. Thus, the effect of Wort and SP6 inhibition are quite different and therefore it is essential analyse these separately. We have now incorporated this inhibitory effect as indicated in page 19, line 452.

R1.13: *Comment 4) The authors need to provide sufficient details so the work is reproducible. For example, what is the cell seeding confluency? I can't find a (supplemental) table listing all the reagents (esp. antibodies). Will the computer codes be publically available?*

Response: We thank the reviewer for bringing this point to our notice. We have now included sufficient experimental details in methods and Supplementary information. The cell-seeding density is specified in the Methods M1, page 27, line 652 in the revised manuscript. S1.1 in S1

Text lists all the reagents and antibodies used. Experimental raw data and codes pertaining to model parameter estimation, simulation and flux analysis are placed in opensource GitHub repository, which is made publicly available at the https://github.com/baichat90/SB-BT-Modulation-of-TNF-mediated-crosstalk-06092022 . We have also placed a ReadMe.doc file as a guide for a reader to these files. We have now included this information in the Data availability statement (page 31, line 757-759 in the revised manuscript).

R1.14: *Comment 5)_Can the authors elaborate on the statement "inhibiting pJNK and pAKT transient dynamics optimizes this accumulated Caspase3 guided apoptotic response"? Being optimal relative to what? How can one tell some optimal results were obtained?*

Response: We thank the reviewer for highlighting the lack of clarity in this statement. We note that pAkt and pJNK transients regulate the caspase3 dynamics and inhibiting these leads to early rise of caspase3 to a high fold change (Figs 5C-vi, 5D-vi) as compared to the control case (Fig. 1C-i). In order to appropriately capture this observation, we have now re-phrased the corresponding sentence in the abstract to improve the clarity. The re-phrased sentence now reads as "Interestingly, perturbing pJNK and pAKT transient dynamics fine-tunes this accumulated Caspase3 guided apoptotic response." in the Abstract of the revised manuscript in page 2, lines 30-31.

R1.15: *5a: Moreover, when performing the three experiments (TNFα, TPL only and TPL + TNFα+ TPL), what states/situations were the cells at? Under stress, starving, or healthy seeding environments? Would these factors interfere with the final cellular fate decisions?*

Response: For all experiments, cells were grown in a natural healthy seeding environment without any serum starvation. All experiments were done with the same protocol (pertaining to cell culture) as specified in the methods (Methods, M1). Since cells were grown under healthy environment without any stress, and culturing conditions were same for all experiments any effect on the cell-fate decision would at best be uniform across all observations.

R1.16: *5b: There are some more specific comments on the writing. The authors should use simple language, and correct typos.*

Response: We have now thoroughly re-written several parts of the manuscript using simple language. For example, we have re-phrased "In our model Bcl2 and PI3K directly activate the pAKT in contrast CAPP inhibit pAKT, TNFR1 and pJNK activate Caspase3, whereas pAKT and NFκB inhibits the Caspase3." to "In our model, Bcl2 and PI3K directly activate pAKT, while CAPP inhibits pAKT. TNFR1 and pJNK activate Caspase3, while pAKT and NFkB inhibits Caspase3." in page 9, line 193-194 in the revised manuscript. We have re-phrased several complex sentences into simple ones throughout the revised manuscript. We have corrected the typos and grammatical errors to the best extent.

R1.17: *5b i) Page 5, paragraph right above "Results" section: The kinetic parameters required for the detailed model considered are usually unavailable.*

Response: We have now re-phrased this sentence to "The parameters involved in the kinetic model needs to be estimated, as these are not available in the literature for the considered cell-type." in page 6, lines 109-111 in the revised manuscript.

R1.18: *5b ii) Page 6, last paragraph: "no significant difference in Caspase3 dynamics"---difference between what? Cells before and after treatment?*

Response: To improve clarity, we have now re-phrased this sentence as "In the case of only TNFa stimulation, Caspase3 dynamics being relatively unaltered for the entire duration (Fig 1Ci, blue), corroborates with the low apoptosis percentage over 24 hours (Fig 1A, blue bar)." in pages 7-8, lines 151-154 in the revised manuscript.

R1.19: *5b iii) Page 8: "In our model Bcl2 and PI3K directly activate the pAKT in contrast CAPP inhibit pAKT"? --Can't understand this sentence.*

Response: We have re-phrased this sentence as "In our model, Bcl2 and PI3K directly activate pAKT, while CAPP inhibits pAKT. TNFR1 and pJNK activate Caspase3, while pAKT and NFkB inhibits Caspase3." in page 9, lines 193-194 in the revised manuscript.

R1.20: *5b iv) Page 8: "These mediating crosstalk between the marker proteins leading to apoptosis regulation were incorporated"---Consider rewording, such as " We incorporated these mediating…."*

Response: We have re-phrased this sentence as "We incorporated these entities into the model to study the cross-talk between the marker proteins leading to apoptosis regulation." in page 9, lines 196-197 in the revised manuscript.

R1.21: *5b v) Fig 2B: Need to make it clear whether the simulated results were obtained from fitting the experimental data, or predicted with parameter values estimated independently from the data. For the former, one should label it clearly as "Model fitting results".*

Response: We thank the reviewer for pointing this lack of clarity. We have now re-labeled the "Simulated data" as "predicted" in Fig 6A.

R1.22: *5b vi) Page 11: One or two sentences explaining reaction flux will be helpful to general readers to get intuitive understanding of the quantity.*

Response: In the paragraph starting "We next focus…" we have now introduced the following sentences explaining reaction flux for have a general reader gain an understanding. "A signal flowing via an interaction from a start to an end node can be quantified by the reaction flux transduced by it. This reaction flux via an interaction is specified by the rate at which the start node influences the dynamics of the end node." in page p11, lines 249-252 in the revised manuscript.

R1.23: *5b vii) Page 11, second paragraph: as its activity is repressed by NFκB.*

Response: We have now replaced "it's" with "its" in page p12, line 273 in the revised manuscript.

R1.24: *5b viii)Fig 5 C &D: It might be clearer to indicate predicted and measured results on the figure.*

Response: We have incorporated the suggestion in Fig. 6

R1.25: ***5b ix) Page 21: There are several parallel pathways that could influence this cell-death (decision?)***

Response: We have now included the word "decision" at the end of the sentence in page 25, line 586 in the revised manuscript.

Reviewer#2

R2.1: *This manuscript utilized an experiment-informed mathematical model to investigate the crosstalk of intracellular signals and Caspase3 activation and for cell apoptosis, as well as how they are mediated by NFκB. The authors demonstrate the predictive power of this model in an independent condition (Figure S9) and when the cells were treated with inhibitors (Figure 5). The authors further used this model to predict cell apoptosis under different inhibitors conditions. Overall, the model was well examined and offered insights into the interplay of intracellular signals. I have several suggestions:*

Response: We thank the reviewer for the appreciation and encouraging words, and for the suggestions. We have incorporated the suggestions.

R2.2: *1) It is not well justified that the correlation between apoptosis and AUCcasp3 (Figure 4) can persist in the same way for different inhibitor conditions and can thus be applied to study other inhibitors, as presented in Figure 6. Can the authors offer some explanations for that?*

Response: Cleavage of caspase3 is linked to nuclear and DNA degradation resulting in apoptosis (Elmore 2007, Schlatter et al, 2009). Therefore, understanding Caspase3 regulation is essential for rationally controlling apoptosis (Thornberry et al, 1998). Thus, Caspase3 activation is an irreversible commitment to apoptotic response. Apoptosis being a phenotypic response, one of the possible ways to connect the apoptotic dynamics with the transient Caspase3 response is to quantify using the accumulated levels of Caspase3. The dynamic signaling cross-talk analysis presented are all between various signaling entities that are upstream of Caspase3. The inhibitory action via Wortmannin or SP6 are on these entities. Therefore, the relation obtained between the accumulated levels of Caspase3 and the apoptosis under non-inhibitory conditions would continue to hold under inhibitory conditions as well. We have incorporated this point in the sentences "(Note that the inhibitory action via Wort or SP6 are on signaling entities upstream of Caspase3. Therefore, the relation obtained between the AUC_{casp3} and apoptosis under non-inhibitory conditions (Fig. 5Biv-vi) would continue to hold under inhibitory conditions as well.)" in page 22, lines 531-533 in the revised manuscript.

R2.3: *2) The data comparison between the model and experiments in Figure 4B is almost identical. I am worried about a potential overfitting of the model. Can more analyses be done to justify the transferability of the model in this case?*

Response: We thank the reviewer for bringing our attention to this important aspect of the relation between accumulated Caspase3 levels and apoptosis. To assess the goodness of fit, we repeated the fitting with two other sets, each having trajectories of entities from 5 independent best fit parameters. The reduced χ^2 values suggest that the fit is good for TNFα treatment case. However, for TPL and TNFα+TPL cases it overfits. We have included this analysis in S7 Text.

As discussed in the response to comment R2.2, the dynamic signaling cross-talk analysis presented are all between various signaling entities that are upstream of Caspase3. The inhibitory action via Wortmannin or SP6 are on these entities. Therefore, the relation obtained between the accumulated levels of Caspase3 and the apoptosis under non-inhibitory conditions is transferable to the inhibitory conditions as well even though the model overfits in some cases. We have incorporated this in the main text in the sentences "(Note that the inhibitory action via Wort or SP6 are on signaling entities upstream of Caspase3. Therefore, the relationship obtained between the AUC_{casp3} and apoptosis under non-inhibitory conditions (Fig. 5Biv-vi) would continue to hold under inhibitory conditions as well.)" in page 22, lines 531-533 in the revised manuscript.

R2.4: *3) In the section "Data-driven mathematical model of TNFα network", the authors mention, "Since TNFR2 is activated only by 26kDa transmembrane TNFα, TNFR2 and its downstream signaling are not considered in the model". More discussion, perhaps in the Discussion and Conclusion section, will be helpful in explaining the exclusion of TNFR2 for regulating cell fate.*

Response: TNFα can be found in two different forms. The 17.3 kDa soluble trimeric molecule stimulates only TNFR1 and the 26 kDa membrane-bound trimeric signals via both TNFR1 and TNFR2 (Wajant et al, 2019). In this study, we considered apoptotic response and the signaling marker dynamics due to stimulation by the soluble form. Therefore, the kinetic model proposed in this study only consists of signaling pathways downstream of TNFR1. We have discussed this in the sentences "The experimental data represents the dynamical response due to activation of signaling pathways downstream of TNFR1 stimulated by the soluble 17.3 kDa TNFα. (Note that signaling due to TNFR2, activated only by 26 kDa transmembrane TNFα, and its downstream signaling are ignored.)." in page 24, lines 576-579 in the revised manuscript.

R2.5: *4) References 2 and 57 refer to the same article.*

Response: We have now removed this redundancy and fixed it.

R2.6: *5) Some figures in SI text are mislabeled, for example, Figure S4 in S2.4.*

Response: We have corrected the figure citations in both main text and Supplementary Text.

R2.7: *6) Some labels are missing in Figure S5.*

Response: We have updated the figure caption with labels explained appropriately. The modified caption of old Fig S5, which is S2 Fig in the revised manuscript is **"S2 Fig**. **Detailed TNFα signaling network**. Activation and inhibitory actions are represented by solid (black) and dashed (red) lines, respectively. Green and orange boxes, respectively represent the inactive and active forms of an entity. The model contains 34 species and 81 parameters including 3 scaling constants." in page 39, lines 1048-1051.

Reviewer #3:

R3.1: *In this manuscript, the authors made both experimental and simulation studies on the optimal apoptotic response, i.e., the modulation of signaling cross-talk between pJNK and pAKT. In the experiments, the authors observed that the inhibitor of NF - kB, TPL, can promotes apoptosis remarkably in the presences or absence of TNF-α. Then, they developed a data-driven model to reveal the mechanism in cellular response to TNF-α. They can reproduce the time courses of the key signaling markers by mathematical modeling. They attempted to unravel the mechanism underlying the crosstalk between the JNK and AKT pathways from different perspectives. Moreover, they found that inhibiting pJNK and pAKT transient dynamics optimizes this accumulated Caspase3-dependent apoptotic response. Their results are very relevant, and also valuable for further study. However, the presentation of the manuscript is not very clear, and should be improved largely, and also some more discussions should be added. Especially, some comments as the following should be considered.*

Response: We thank the reviewer for the observations, comments and suggestions. We have addressed all the comments, and thoroughly revised the manuscript to improve the presentation and clarity.

R3.2: *1) NF-kB was mainly considered as a prosurvival factor in this work. The time courses of NF-kB or its targets should be shown experimentally in different cases to reflect the activities of NF-kB. The comparison of NF-kB activities is helpful of validating the inhibitory effects of TPL on NF-kB. Moreover, similar comparison is also required in the simulation results.*

Response: We thank the reviewer for suggesting this point. While we were unable to directly estimate the NFκB activity, in order to quickly assess the effect of TPL, which is a well-established specific inhibitor of NFκB transactivation [reference 49 in the revised manuscript], we quantified TPL dose dependent apoptotic response (S1 Fig - A, pasted below). This dose-dependency clearly shows strong apoptosis response for 60 nM TPL concentration indicating that the NFκB pro-survival signals in ~80% of cells are indeed shutdown. This indirectly suggests that the activity of NFκB is severely hampered at 60 nM TPL concentration.

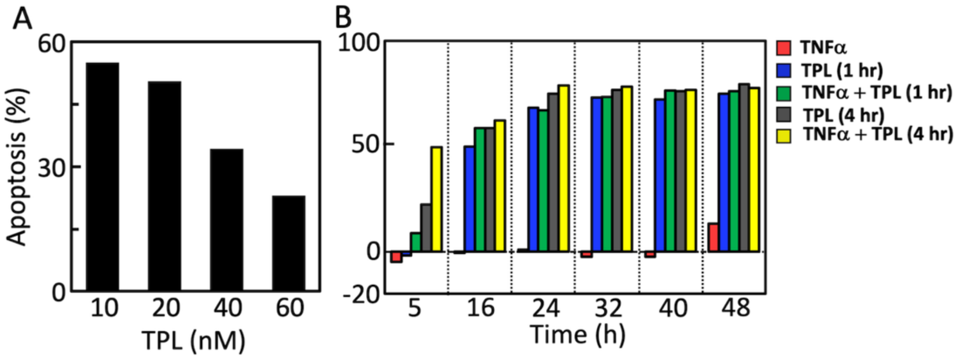

S1 Fig. Effect of TPL on cell survival. (A) Dose-response of TPL on cell-survival. (B) Effect of TPL pre-treatment duration on apoptosis. Note that negative Apoptosis % in (B) for a few cases are due to negative control (only cells) being more than that when treated.

We further complemented experimental observations with model analysis to understand the effect of NFκB activity under TPL conditions. Model simulations show that inhibition of NFκB by TPL cannot be restored even in the presence of a combination of TPL and TNFα (S7 Fig, pasted below). This further corroborates that TPL inhibition is indeed significant in shutting down the activity of NFκB. This also highlights the cause of higher cell-death experimentally observed under only TPL condition. The flux analysis further substantiates the key role played by TPL mediated NFκB inhibition (Fig. I in S4 Text, pasted below). We have discussed the importance of TPL mediated NFκB inhibition in the revised manuscript in the sentences "However, in the

presence of TPL, the inhibition by XG appears to be less effective (Fig 3Bii and 3Cii), which helps to stimulate the dynamics of pJNK at early time points (Fig 2Bv and 2Bviii).” in page 13, lines 291-293 and “Under the influence of TPL in the absence (Fig 3Biii) and presence (Fig 3Ciii) of TNFα, the contribution from NFκB reduced significantly, while pAKT plays a predominant role in maintaining the dynamics of Caspase3 (Fig 2Bix).” In page 13, lines 302-305.

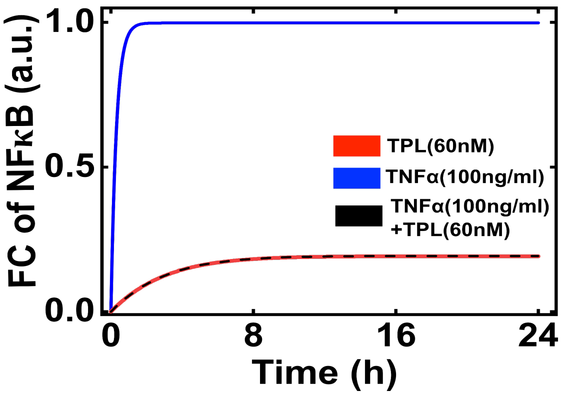

S7 Fig. NFκB dynamics under different stimulation conditions. Trajectories of NFκB protein obtained by simulating the model with best-fit parameter set (S4 Table).

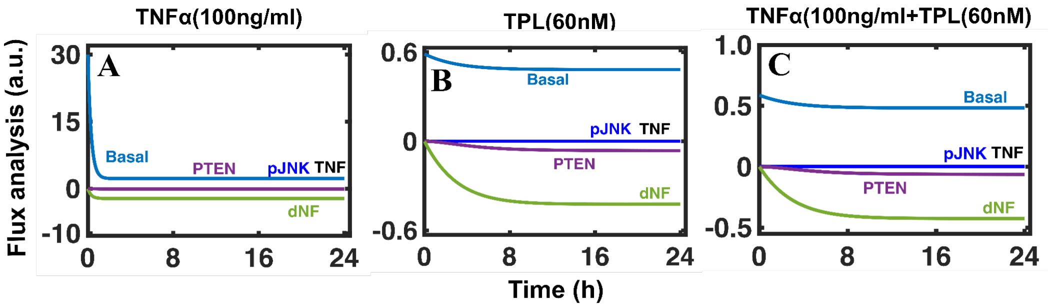

Fig I (S4 Text). Temporal evolution of fluxes contributing to the dynamics of NFκB under the three stimulation conditions. Expression for these fluxes are provided in S4 Table.

R.3.3: *2) According to their model (Fig. 2A), it was assumed that JNK can activate NF-kB. How does this regulation influence apoptosis induction in the presence NF-kB inhibition by TPL.*

Response: We thank for bring to our attention this interesting aspect about cross-talk regulation by JNK. The flux analysis around NFκB (Fig I in S4 Text) shows that pJNK mediated NFκB activation is weak at all time points, which is the case even under only TNFα or TNFα+TPL cases.

This indicates that the JNK mediated NFκB activation in the presence of TPL will not play a significant role in regulating apoptosis.

R3.4: *3) The dose of TNF-α was fixed at 100ng/ml. How does the doses of TNF-α modulate the crosstalk between signaling pathways and apoptosis induction?*
Response: We thank the reviewer for raising this question. We performed model simulations for at different TNFα doses with and without TPL treatment. The model simulated trajectories demonstrate that Caspase3, pJNK and pAKT transients is similar for a wide range of TNFα dose which encompasses the chose stimulation concentration of 100 ng/ml. This point is discussed in the sentences "Model simulations show that inhibition of NFκB by TPL cannot be restored even in the presence of a combination of TPL and TNFα (S3 Text, S6 Fig, S7 Fig) which substantiates the similarity in the transient Caspase3 dynamics for TPL and TNFα+TPL treatments (Fig. 2Bvi., 2Bix). This further shows that TPL inhibition is indeed significant in shutting down the activity of NFκB." in page 10, lines 226-230 in the revised manuscript.

R3.5: *4) In Figs. 5C and 5D, there exists obvious bias between the simulation and experimental results for pJNK, the authors should give an explanation to this point.*
Response: We thank the reviewer for raising this issue. We revisited the model predictions under Wortmannin and SP6 inhibitory conditions (Figs. 5C and 5D), as has also been suggested by Reviewer 1 (comment **R1.11** above) and noted that Wortmannin can also inhibit the activation of XG by NFκB (Manna and Aggarwal, 2000 [reference 67 in the revised manuscript]). The newly introduced branch analysis (Fig 4) revealed that the dynamics of pJNK is dictated, among others, by a few nested positive and negative feedback loops involving RAF and pERK. Unfortunately, the model-based parameter estimation did not offer a good estimate of the kinetic parameters such as K_{mae}, K_{eir}, K_{air} (S9 Fig) which control the flux towards pJNK from these nested feedback loops working via RAF and pERK. Therefore, the dynamic state of the pJNK is poorly represented during the model parameter estimation which explains the observed discrepancy in the model prediction for pJNK under inhibitory conditions. This point is now discussed briefly in the sentences "The nature of synergistic dynamics observed in B_{nj4} indicates a weak representation of this branch in the overall pJNK transient. This is due to the poor estimation of the kinetic parameters (such as K_{mae}, K_{eir}, and K_{air}) involved in the RAF-ERK-JNK feedback loop, which

could explain the qualitative difference between the model fitting and experimental observations for pJNK under TNFα+TPL condition." in page 16, lines 366-370 in the revised manuscript.

R3.6: *5) The duration of two treatments in the combinatorial therapies may results in distinct cellular outcomes (PMID: 26965628). How does the duration between TPL pre-treatment and TNF-α exposure influence cell fates?*

Response: We thank the reviewer for bringing our attention to the important of pre-treatment duration of combinatorial therapies leading to different cellular outcome and also pointing to the reference Chen et al., Science, 2016. We assessed the extent of cell-death by varying the duration between TPL pre-treatment and TNFα exposure. We present this in S1 Fig-B, pasted below. Increasing the duration of pre-treatment from 1 to 4 hours, followed by a 24 h TNFα exposure leads to a marginal change of 10% additional cell-death.

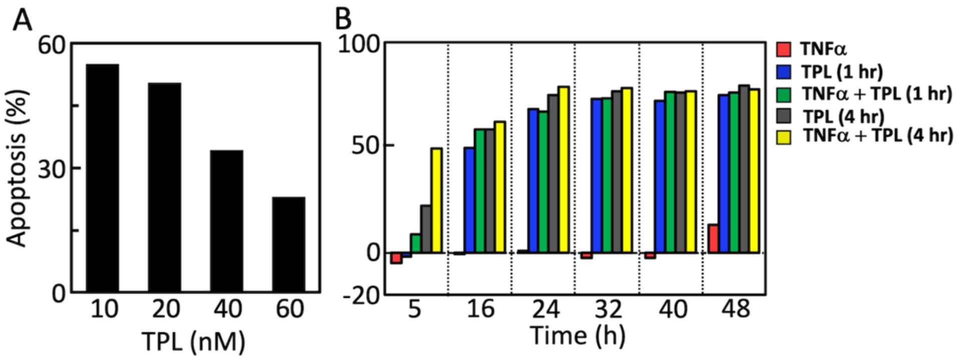

S1 Fig. Effect of TPL on cell survival. (A) Dose-response of TPL on cell-survival. (B) Effect of TPL pre-treatment duration on apoptosis. Note that negative Apoptosis % in (B) for a few cases are due to negative control (only cells) being more than that when treated.

R3.7: *6) In this work, the marker proteins showed different regulation modes in the early and late phase of the response. In the DNA damage response, p53 also exhibits different regulation effects on apoptosis in the early and late phase of the response (PMID: 29048401; PMID: 21576488; PMID: 19617533). The shared principles in cellular response to different stimuli should be discussed.*

Response: We thank the reviewer for bringing our attention to the feature of different early and late phase in another signaling framework. We have now discussed this commonality in the early

and late phase responses reported in our study and those in the p53 regulated DNA damage response in the sentences “Such a different early and late-phase responses of the marker proteins have also been observed during p53 signaling induced by DNA damage response [77-80]” in page 26, lines 618-619 in the revised manuscript.

R3.8: *7) The formats of the items in the Reference should be unified.*

Response: We have now thoroughly checked the formatting of the references and unified the same.

R3.9: *8) The English writing of the manuscript should be improved. For example, page 10, the bottom paragraph, “is” in “how the overall dynamics is regulated” should be corrected; page 15, the sentence “which is only moderately affected by ……” should be removed to page 14 to follow closely with the last sentence; page 21, the second line from the bottom, “connect” should be connection. Of note, the list is not exhausted, the authors should check the writing of the manuscript entirely.*

Response: We have now revised these sentences and also several others in the manuscript to improve the overall writing and clarity. We replaced the word “connect” to “connection” in page 26, line 627 in the revised manuscript. We simplified the complex sentences into multiple simple ones in several places. For e.g., we rephrased a complex sentence in to the following two sentences “The underlying TNFα molecular network contains several integrated pathways and multiple feedback regulations. Both quantitative and qualitative mathematical models of the network could provide a precise understanding of the dynamical properties of such a complex system.” in page 5, lines 87-90 in the introduction of the revised manuscript.
